# Nuclear and Nucleolar Localization of Bovine Adenovirus-3 Protein V

**DOI:** 10.3389/fmicb.2020.579593

**Published:** 2021-01-06

**Authors:** Xin Zhao, Suresh K. Tikoo

**Affiliations:** ^1^VIDO-InterVac, University of Saskatchewan, Saskatoon, SK, Canada; ^2^Department of Veterinary Microbiology, University of Saskatchewan, Saskatoon, SK, Canada; ^3^Vaccinology and Imuunothepapeutics Program, School of Public Health, University of Saskatchewan, Saskatoon, SK, Canada

**Keywords:** BAdV-3, pV, NLS, NOLS, capsid, protein-protein interaction

## Abstract

The L2 region of bovine adenovirus-3 (BAdV-3) encodes a *Mastadenovirus* genus-specific protein, designated as pV, which is important for the production of progeny viruses. Here, we demonstrate that BAdV-3 pV, expressed as 55 kDa protein, localizes to the nucleus and specifically targets nucleolus of the infected cells. Analysis of deletion mutants of pV suggested that amino acids 81–120, 190–210, and 380–389 act as multiple nuclear localization signals (NLS), which also appear to serve as the binding sites for importin α-3 protein, a member of the importin α/β nuclear import receptor pathway. Moreover, pV amino acids 21–50 and 380–389 appear to act as nucleolar localization signals (NoLs). Interestingly, amino acids 380–389 appear to act both as NLS and as NoLS. The presence of NoLS is essential for the production of infectious progeny virions, as deletion of both NoLs are lethal for the production of infectious BAdV-3. Analysis of mutant BAV.pVd1d3 (isolated in pV completing CRL cells) containing deletion/mutation of both NoLS in non-complementing CRL cells not only revealed the altered intracellular localization of mutant pV but also reduced the expression of some late proteins. However, it does not appear to affect the incorporation of viral proteins, including mutant pV, in BAV.pVd1d3 virions. Further analysis of CsCl purified BAV.pVd1d3 suggested the presence of thermo-labile virions with disrupted capsids, which appear to affect the infectivity of the progeny virions. Our results suggest that pV contains overlapping and non-overlapping NoLS/NLS. Moreover, the presence of both NoLS appear essential for the production of stable and infectious progeny BAV.pVd1d3 virions.

## Introduction

Bovine adenovirus-3 (BAdV-3), a member of the *Mastadenovirus* genus, is a non-enveloped icosahedral particle, which contains a double-stranded DNA genome of 34,446 bp organized into early, intermediate, and late regions (Reddy et al., 1998). Despite its similarity in genome organization with human adenovirus-5 (HAdV-5), BAdV-3 appears to possess certain distinct features (Reddy et al., 1998; Idamakanti et al., 1999; Xing et al., 2003; Bangari and Mittal, 2006; Xing and Tikoo, 2006, 2007), including the organization of late (L) transcriptional unit into seven (L1–L7) regions, in contrast to HAdV-5 (Reddy et al., 1998).

The L2 region of HAdV-5 encodes a minor capsid protein named protein V (pV), which associates with the viral genome and bridges the core and the capsid proteins (Vayda et al., 1983; Chatterjee et al., 1985; Matthews and Russell, 1998; Lehmberg et al., 1999). pV appears to be essential for virus replication in primary cells, but not in cancerous cells (Ugai et al., 2007). pV localizes to nuclei using monopartite\bipartite NLS (Matthews, 2001; Hindley et al., 2007), multiple import factors, and to the nucleolus using multiple NoLs and a transportin dependent import pathway (Hindley et al., 2007). Although expression of pV does not alter the localization of nucleolar proteins (B23, nucleolin) in infected cells, the over expression of pV redistributes nucleolin and nucleophosmin to the cytoplasm in transfected cells (Matthews, 2001). While sumoylation of pV alters the adenovirus replication, it does not change the nucleolar localization of pV in infected cells (Freudenberger et al., 2018).

The nuclear localization of a protein is a well-characterized process regulated by nuclear pore complexes (NPCs) and requires active transport mechanisms, including nuclear transport receptor proteins and specific nuclear localization signal (NLS) sequences, on the transported viral protein. Unlike the nucleus, the nucleolus is a membrane-free sub nuclear structure involved in ribosome biogenesis, cell cycle regulation, cellular stress response, apoptosis, and viral replication (Salvetti and Greco, 2014). Nucleolar localization depends on the interactions of nucleolar constituents (proteins or rRNA) with specific viral protein sequences usually rich in arginine and lysine, which can act as the nucleolar localization signals (NoLS; Reed et al., 2006).

Recently, we reported on the characterization of late structural\non-structural BAdV-3 proteins and demonstrated that, while BAdV-3 52K (Paterson et al., 2012), pVIII (Ayalew et al., 2014), 22K (Said et al., 2018), and IVa2 (Woldemariam et al., 2020) utilize the importin α/β pathway to localize to the nucleus in infected cells, BAdV-3 33K utilizes both importin α/β and transportin -SR nuclear import receptor pathways for localization to the nucleus in infected cells (Kulshreshtha et al., 2014).

The L2 region of the late transcription unit of BAdV-3 encodes pV, which is collinear with pV of HAdV-5 (Reddy et al., 1998) and appears essential for the production of infectious progeny virions (Zhao and Tikoo, 2016). A recent report suggested that pV interacts with 33K in BAdV-3 infected cells (Kulshreshtha and Tikoo, 2008). Although homologs of pV have been identified in other members of *Mastadenovirus* genus, BAdV-3 pV show 28–41% amino acid identity with pV proteins of other *Mastadenoviruses* (Reddy et al., 1998). Since there appears reasonable variation in similarity among pV encoded by other members of *Mastadenovirus*, we sought to characterize this protein in detail. Here, we report the characterization and identification of signals mediating nuclear and nucleolar localization of BAdV-3 pV and demonstrate that NoLs of pV are important for BAdV-3 replication.

## Results

### Expression of pV During BAdV-3 Infection

**FIGURE 1:**
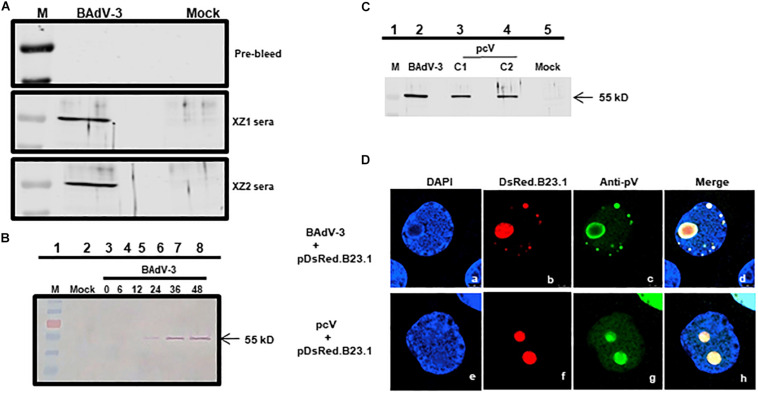
Expression of pV. Proteins from BAdV-3 infected MDBK cells **(A,B)** or indicated plasmid DNA transfected cells (**C**; lanes 3 and 4) or mock infected/transfected cells were harvested at different time points, separated by SDS-PAGE, and transferred to nitrocellulose membranes. The separated proteins were probed by Western blot using anti-pV serum. The position of the molecular weight marker (lane M) in kD was used for sizing the protein bands. **(D)** CRL cells were transfected with plasmid pDsRed.B23 DNA and infected by BAdV-3 (panels a–d) or co-transfected with plasmid pcV and pDsRed.B23.1 DNA (panels e–h) and fixed at 24 h post-infection\transfection. The DsRed.B23.1 was visualized by direct fluorescence microscopy (panels b, f). BAdV-3 pV was visualized by indirect immunofluorescence microscopy (panels c, g) using anti-pV antiserum and Alexa Fluor 488-conjugated goat anti-rabbit IgG. The nuclei were stained with DAPI.

To characterize BAdV-3 pV, peptides ZX1 (^1^MASSRLIKEEMLDIVAPEIY KRKR^24^) and peptide ZX2 (^180^SRKRGVGKVEPTIQVLASKK RRMA^212^) were synthesized based on their hydrophilicity score (Kyte and Doolittle, 1982) and used to generate anti-pV sera designated as XZ1 and XZ2 sera, respectively. The specificity of the sera was analyzed by Western blot using BAdV-3 infected MDBK cells (ATCC CCL22). As seen in Figure 1A, both XZ1 serum and XZ2 serum detected a protein of 55 kDa in BAdV-3 infected cells. No such protein could be detected in mock-infected cells using XZ1 or XZ2 sera or BAdV-3 infected cells using pre-bleed sera. The protein could be detected at 24–48 h post infection (Figure 1B, lanes 6–8) but not at 12 h post infection (Figure 1B, lane 5). Similarly, anti-pV pooled sera detected a 55 kDa protein in HEK293T cells (ATCC 11268) transfected with plasmid pcV DNA (Figure 1C, lanes 3–4). No such protein could be detected in control plasmid pcDNA3 DNA (mock) transfected cells (Figure 1C, lane 5). The BAdV-3 pV expressed as 55 kDa protein appears between 12–24 h post-infection and could be detected up till 48 h post BAdV-3 infection.

### Subcellular Localization of pV

To determine the subcellular localization of pV, CRL cells (Papp et al., 1997) were transfected with plasmid pDsRed.B23.1 DNA (Gomez and Archambault, 2009) and infected with BAdV-3 at 48 h post-transfection. At 24 h post-infection, the cells were analyzed by indirect immunofluorescence assay using anti-pV serum. As seen in Figure 1D, pV co-localized predominantly with nucleolar protein B23.1 fused to DsRed (pDsRed.B23.1) in BAdV-3 infected cells, suggesting that pV localizes in the nucleolus of the virus infected cells.

To determine if nucleolar localization is dependent on other viral proteins, we determined the localization of pV in Vero cells (ATCC – CCL81), co-transfected with plasmid pcV and pDsRed.B23.1 DNAs by florescence microscopy. As seen in Figure 1D, pV co-localizes predominantly with nucleolar marker B23.1 fused to DsRed in the nucleolus of the co-transfected cells. These results suggest that pV is almost exclusively detected in the nucleolus of the BAdV-3 infected or transfected cells in the absence of any other viral proteins.

### Identification of pV Nucleolar Localization Signal

**FIGURE 2:**
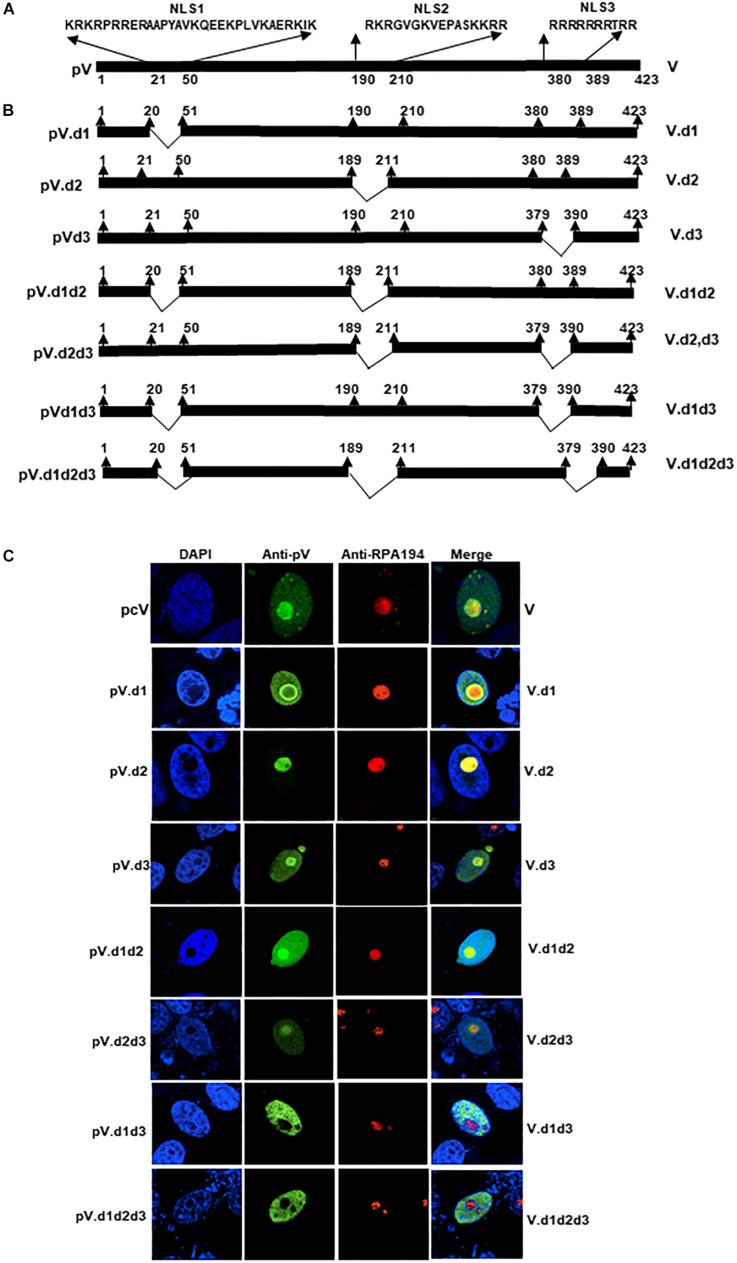
Analysis of BAdV-3 pV nucleolar/nuclear localization signals. **(A)** Schematic representation of BAdV-3 pV. The thick black line represents BAdV-3 pV. The numbers below represent the amino acids of pV. Potential nuclear localization signal (NLS) sequences are depicted. The name of the plasmid is depicted on the left. The name of the protein is depicted on the right. **(B)** Schematic diagram represents mutant pV. Thick black lines represent pV gene, thin black lines represent the deleted regions. The name of the plasmid is depicted on the left. The name of the protein is depicted on the right. **(C)** Sub cellular localization of wild-type pV and pV mutants. Vero cells were transfected with those plasmids expressing wild-type pV and mutant pV genes individually and fixed with 4% formaldehyde at 48 h post-transfection. BAdV-3 pV was visualized by indirect immunofluorescence using anti-pV antiserum and Alexa Fluor 488-conjugated goat anti-rabbit IgG (Jackson Immunoresearch). Nuclei were stained with DAPI and nucleoli were visualized with indirect immunostaining by using RPA194 antibody (C-1; Santa Cruz Biotechnology) and TRITC-conjugated goat anti-mouse IgG (Jackson Immuno-research). The name of the plasmid is depicted on the left. The name of the protein is depicted on the right.

Bioinformatic analysis of the pV protein sequence using motif prediction algorithms such as “Predict Protein” (Yachdav et al., 2014) predicted that the amino acids ^21^KRKRPRRERAAPYAVKQ EEKPLVKAERKIK^50^, ^190^RKRGVGKVEPTIQVLA SKKRR^210^, and ^380^RRRRRRRTRR ^389^ of BAdV-3 pV may act as potential NLSs (Figure 2A). To determine if these domains act as NLSs, we constructed plasmids expressing mutant pV containing specific NLS domain deletions (Figure 2B). Vero cells co-transfected with plasmid pDsRed.B23.1 DNA and individual plasmid DNA expressing mutant pV protein were analyzed by immunofluorescence assay at 48 h post transfection. As seen in Figure 2C, the wild-type mutant localized both in the nucleus and nucleolus of transfected cells. Similarly, the mutant pV containing deletion of amino acid 21–50 (V.d1) or mutant pV containing deletion of amino acid 380–389 (V.d3) localized both in the nucleus and the nucleolus of the transfected cells. However, mutant pV containing deletion of amino acid 190–210 (V.d2) localized in the nucleolus of the transfected cells. Interestingly, mutant pV containing a deletion of amino acids 21–50 and 190–210 (V.d1d2), or deletion of amino acids 190–210 and 380–389(V.d2d3) could be detected in the nucleus and nucleolus of the transfected cells. In contrast, mutant pV containing the deletion of amino acids 21–50 and 380–389 (V.d1d3), or deletion of amino acids 21–50,190–210, and 380–389 (V.d1d2d3), localized predominantly in the nucleus of the transfected cells. These results suggest that amino acids 21–50 and 380–389 act as NoLSs.

**FIGURE 3:**
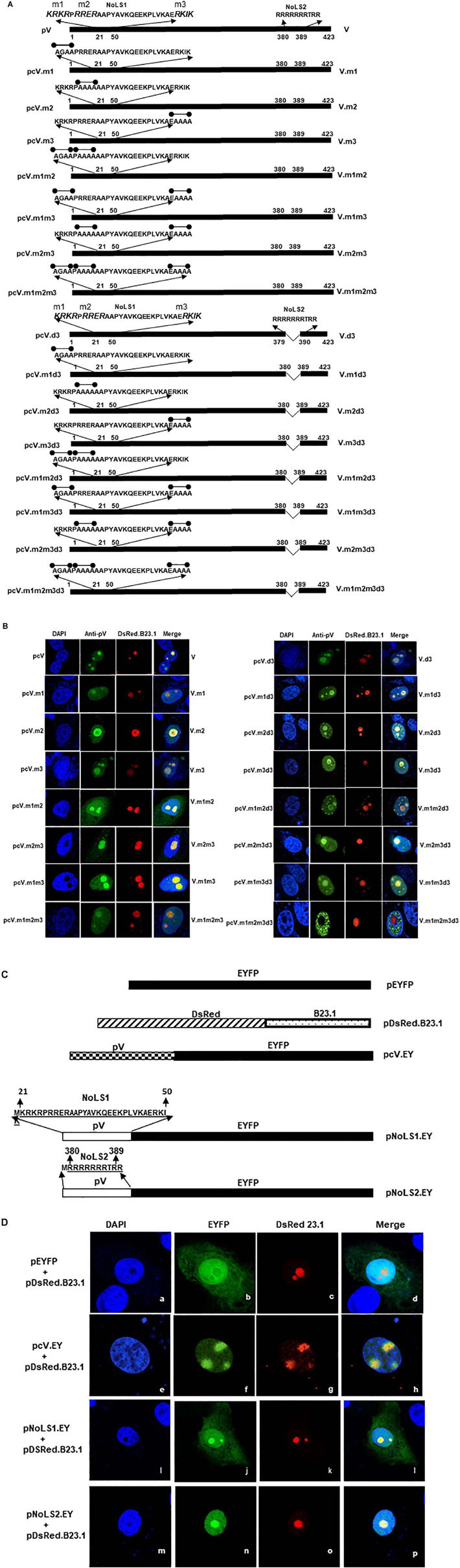
Mutation analysis of pV NoLS1. **(A)** Schematic representation of BAdV-3 pV depicting the amino acid sequence of NoLS1 and NoLS2. The thick line represents the BAdV-3 pV gene. The thin line represents the deleted region. The basic residue rich motifs (m1, m2, and m3) are shown in different font sizes. The mutations are indicated by a thin line with black-filled circles. The numbers above represent the amino acid of BAdV-3 pV. The name of the plasmids is depicted on the left of the panel. The name of the protein is depicted on the right of the panel. **(B)** Sub cellular localization of pV mutants. Vero cells were co-transfected with plasmid pDsRed.B23 and individual indicated plasmid DNAs. At 48 h post-transfection, cells were fixed with 4% formaldehyde. BAdV-3 wild-type and pV NoLS1 mutant proteins were visualized by indirect immunofluorescence microscopy using anti-pV antiserum and Alexa Fluor 488-conjugated goat anti-rabbit IgG (Jackson Immunoresearch). The DsRed.B23.1 was visualized by direct fluorescence microscopy. Nuclei were stained with DAPI. The name of the plasmids is depicted on the left of the panel. The name of the protein is depicted on the right of the panel. **(C)** Schematic representation of fusion proteins containing BAdV-3 pV NoLSs. The DsRed is represented by (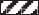). The pV is represented by (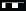). The dotted box represents B23.1. The white box represents BAdV-3 pV nucleolar localization signals amino acids 21–50 or 380–389. The black box represents the EYFP gene. The numbers above represent amino acids of BAdV-3 pV. **(D)** Sub cellular localization of EYFP-fusion protein. Vero cells were co-transfected with individual indicated plasmids expressing EYFP, cV.EY, or EYFP-NoLS fusion proteins and pDsRed.B23.1 DNAs, and fixed with 4% formaldehyde at 48h post-transfection. The cV.EY (panel f), DsRed.B23.1 (panels c, g, k, and o), EYFP (panel b), NoLS1.EY (panel j), and NoLS2.EY (panel n) were visualized by direct fluorescence microscopy. Nuclei were stained with DAPI (panels a, e, i, and m). Merge (panels d, h, l, and p).

Earlier, Weber et al. (2000) suggested that the basic amino acid rich sequence K/R-K/R-X-K/R, wherein X stands for any amino acids that may play a role in the nucleolar localization of a protein. Our analysis of NoLS1 (amino acid 20–50) and NoLS2 (amino acid 380–389) sequence identified three motifs (^21^KRKR^24^, ^26^RRER^29^, and ^47^RKIK^50^) in NoLS1, which have the potential to act as NoLS (Figure 3A). To determine the role of each motif (m1, m2, or m3; Figure 3A) in the nucleolar localization of protein V, we used plasmid pcV.d3 (Figure 3A) DNA containing the deletion of amino acids 380–389 as a plasmid backbone to construct additional plasmids expressing mutant pV proteins in which the basic residues of identified potential NoLS motifs were replaced with glycine\alanine residues (Figure 3A). Vero cells were co-transfected with plasmid pDsRed.B23.1 and individual plasmid DNA expressing mutant pV protein and analyzed with immunofluorescence assay using anti-pV sera. As seen in Figure 3B, pV is predominantly localized in the nucleolus of cells transfected with plasmids expressing wild-type pV or mutant pV protein containing amino acid substitution in a single motif (V.m1, V.m2, and V.m3), double motif (V.m1m2, V.m2m3, and V.m1m3), triple motif (V.m1m2m3), single motif and d3 deletion (V.m1d3, V.m2d3, or V.m3d3), or double motif and d3 deletion (V.m1m2d3, V.m2m3d3, or V.m1m3d3). In contrast, pV is predominantly localized in the nucleus of the cells transfected with plasmids expressing mutant pV protein containing basic amino acid substitution in all three potential NoLS motifs and d3 deletion (V.m1m2m3d3). Moreover, the V.m1m2m3d3 protein appears as multi-punctuate granular dots in the nucleus of transected cells. These results suggested that all three basic residue motifs of NoLS1 have a redundant function in localizing pV to the nucleolus.

To confirm the nucleolar localization function of BAdV-3 pV amino acids 21–50 and 380–389, DNA fragments encoding amino acids 21–50 and 380–389 were fused in-frame with enhanced yellow fluorescent protein (EYFP) gene to create plasmids pNoLS1.EY and pNoLS2.EY expressing fusion proteins (Figure 3C), respectively. Vero cells co-transfected with plasmid pDsRed.B23.1 DNA and either plasmid pNoLS1.EY DNA or plasmid pNoLS2.EY DNA were analyzed by confocal microscopy at 48 h post transfection. As seen in Figure 3D, EYFP protein could be detected in the cytoplasm and nucleus of transfected cells (Figure 3D, panel b). As expected, pV fused to EYFP was detected both in the nucleus and the nucleolus of the transfected cells (Figure 3D, panel f). In contrast, EYFP-fused NoLS1 (amino acids 21–50) was detected in the nucleolus, nucleus, and cytoplasm of the transfected cells (Figure 3D, panel j) and EYFP fused to NoLS2 (amino acid 380–389) was detected in the nucleus and nucleolus of transfected cells (Figure 3D, panel n).

### Identification of pV Nuclear Localization Signal

**FIGURE 4:**
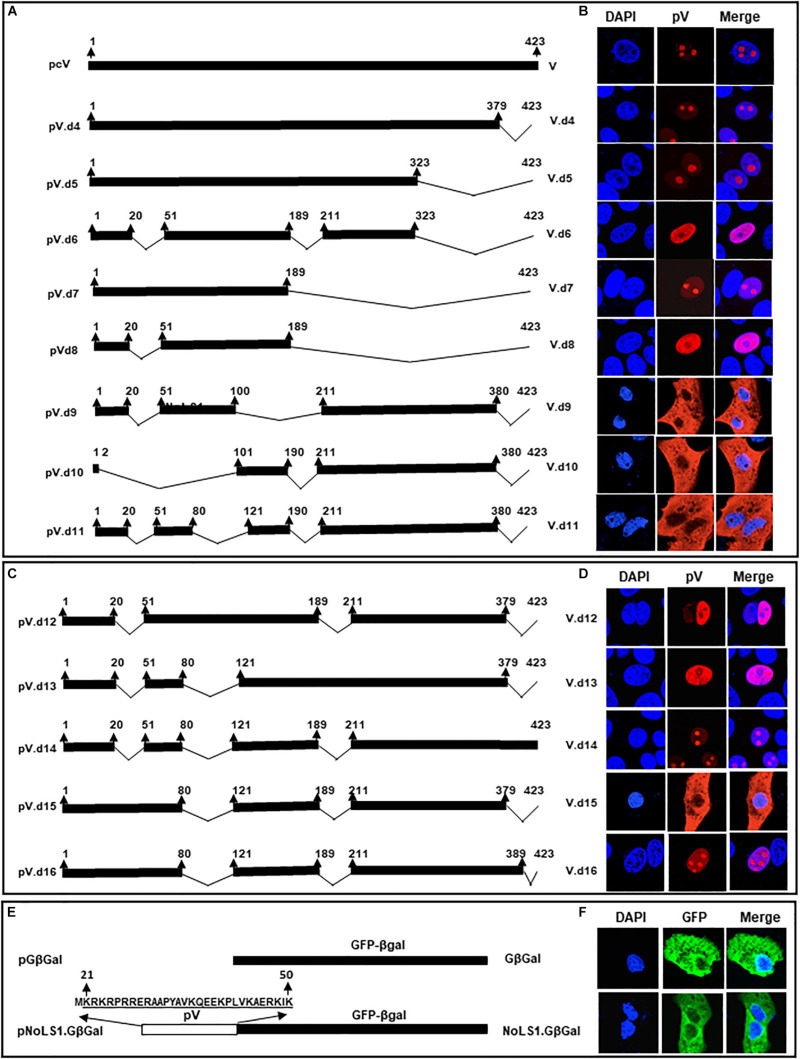
Analysis of BAdV-3 pV nuclear localization signals. **(A)** Schematic representation of wild-type and mutant pV. The thick black line represents BAdV-3 pV gene. Thin black line represents the deleted regions. The numbers above represent amino acids of BAdV-3 pV. The name of the plasmid is depicted on the left. The name of the protein is depicted on the right. **(B)** Sub cellular localization of wild-type pV and pV mutants. Vero cells were transfected with individual indicated plasmid DNA and fixed with 4% formaldehyde at 48 h post-transfection. BAdV-3 wild-type pV and pV mutants were visualized by immunofluorescence using anti-pV antiserum and TRITC-conjugated goat anti-rabbit IgG (Jackson Immunoresearch). Nuclei were stained with DAPI. **(C)** Schematic representation of pV mutants. The thick black line represents BAdV-3 pV gene. Thin black line represents the deleted regions. The numbers above represent the amino acids of BAdV-3 pV. The name of the plasmid is depicted on the left. The name of the protein is depicted on the right. **(D)** Sub cellular localization of pV mutants. Vero cells were transfected with individual indicated plasmid DNA and fixed with 4% formaldehyde at 48 h post-transfection. BAdV-3 pV mutants were visualized by immunofluorescence using anti-pV antiserum and TRITC-conjugated goat anti-rabbit IgG (Jackson Immunoresearch). Nuclei were stained with DAPI. **(E)** Schematic representation of GFP-βGal fusion protein containing BAdV-3 pV NoLS1. The white box represents BAdV-3 pV NoLS1 amino acids 21–50. The black box represents the fusion protein GFP-βGal. The numbers above represent amino acids of BAdV-3 pV. The name of the plasmid is depicted on the left. The name of the protein is depicted on the right. **(F)** Sub cellular localization of fusion protein NoLS1-GFP-βGal. Vero cells were transfected with those plasmids individually and fixed with 4% formaldehyde at 48 h post-transfection. The fusion protein GFP-βGal was visualized with direct fluorescence microscopy. Nuclei were stained with DAPI.

To determine the NLSs of BAdV-3 pV, we constructed plasmids expressing mutant BAdV-3 pV containing truncations and \or internal deletions (Figure 4A). Vero cells were transfected with individual recombinant plasmid DNA. At 48 h post transfection, transfected cells were analyzed by indirect immunofluorescence using anti-pV sera. As seen in Figure 4B, mutant pV proteins containing deletions of amino acids 191–423 (V.d4, V.d5, V.d6, and V.d7) appear to be localized in the nucleus of the transfected cells. Moreover, mutant pV proteins containing deletion of amino acid 21–50 and 190–423 (V.d8) also localized to the nucleus of the transfected cells. As expected, V.d4, V.d5, and V.d7 (containing one or both identified NoLSs) localized to the nucleolus of the transfected cells, while V.d6 (with the absence of identified NoLS1 and NoLS2) localized to the nucleus of the transfected cells. In contrast, analysis of mutant V.d9 protein (containing deletion of amino acids 21–50 + 101–210 + 380–423), mutant V.d10 protein (containing deletion of amino acid 2–100 + 190–210 + 380–423), and mutant V.d11 protein (containing deletion of amino acids 21–50 + 81–120 + 190–210 + 380–423) suggested that amino acids 21–50, 81–120, 190–210, and 380–423 might contain NLSs motifs, which may have a redundant function.

Since it appeared that multiple NLSs are involved in pV localization to the nucleus, we combined identified deletions (Figure 4B) to determine the requirement/redundancy of the multiple NLSs. To this end, we constructed plasmids (Figure 4C) containing deletions of three of the four regions of pV containing potential NLSs. Vero cells were transfected with individual plasmid DNAs and subcellular localization of mutant pVs proteins were analyzed at 48 h post transfection by indirect immunofluorescence using anti-pV sera. As seen in Figure 4D, mutant pV proteins containing amino acids 81–120 (V.d12) and 190–210 (V.d13) or amino acid 380–423 (V.d14) localized to the nucleus\nucleolus. Mutant V.d16 protein retaining amino acid 380–389 (potential NLS) with deletion of potential NLSs (amino acids 81–120, 190–210) was localized in the nucleus and nucleolus of transfected cells. In contrast, mutant pV protein (V.d15) retaining amino acid 21–50 (potential NoLS) with deletion of all potential NLSs (amino acids 80–120, 190–211, and 379–423) are localized predominantly in the cytoplasm of the transfected cell. Analysis of mutant V.d15 suggested that NoLS1 (Kyte and Doolittle, 1982; Lafemina et al., 1989; Papp et al., 1997; Kohler et al., 1999; Weber et al., 2000; Matthews, 2001; Zhou et al., 2001; Cheng et al., 2002, 2013; Kulshreshtha et al., 2004, 2014; Ladd and Cooper, 2004; Sheng et al., 2004; Wu et al., 2004; Cros et al., 2005; Olson and Dundr, 2005; Stracker et al., 2005; Wodrich et al., 2006; Tollefson et al., 2007; Kulshreshtha and Tikoo, 2008; Gomez and Archambault, 2009; Du and Tikoo, 2010; Paterson, 2010; Scott et al., 2010; Blanchette et al., 2013; Boisvert et al., 2014; Yachdav et al., 2014; Makadiya et al., 2015; Musinova et al., 2015; Zhao and Tikoo, 2016) is not sufficient to localize the pV (containing NLSs deletions) to the nucleus on its own. These results suggested that amino acids 380–389 contain NLS.

To examine if pV NoLSs can serve as NLSs, plasmids expressing pV.d16 (containing deletion of potential NLS motifs amino acids [81–120 + 190–210] but retaining potential NLS\NoLS motif amino acid 380–389; Figure 4C) and plasmid pNoLS1-GFPβGal (NoLs1 fused to cytoplasmic protein GFPβGal (Figure 4E) were constructed and used to transfect Vero cells. As shown in Figures 4D,F, mutant V.d16 protein and fusion protein NoLS1-GFPβGal fusion were localized in the nucleus/nucleolus and cytoplasm of transfected cells, respectively. Compared to V.d15, V.d16 proved that the presence of 380–389 (NoLS2) can locate pV to the nucleus and nucleolus. These results suggest that, while NoLS1 can localize pV to the nucleolus but not nucleus, the NoLS2 localizes pV to both the nucleus and nucleolus.

### Interaction of pV With Importins

**FIGURE 5:**
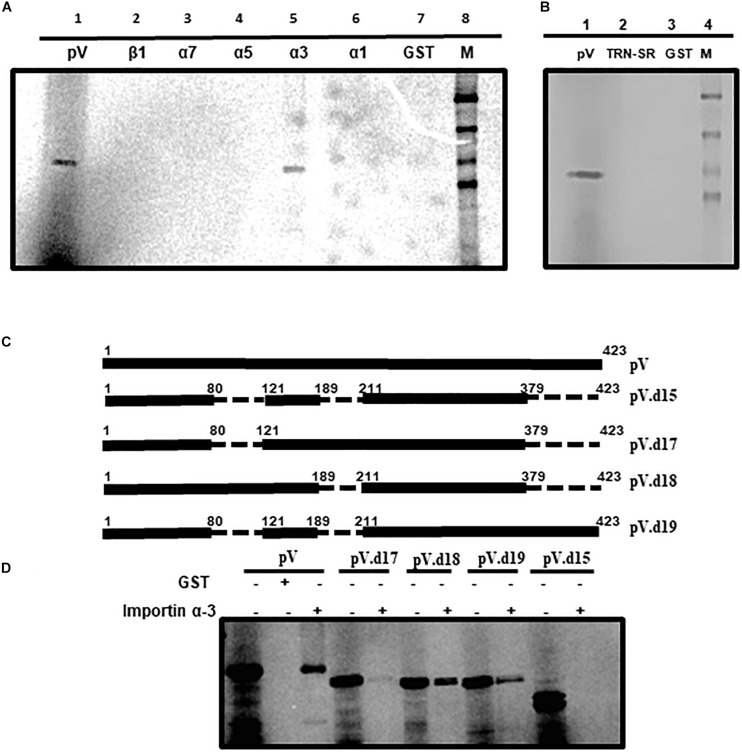
Interaction of pV with transport receptors. **(A)** *In vitro* interaction of pV with importin α3. *In vitro* synthesized and [^35^S]-labeled BAdV-3 pV was incubated with purified GST fusion proteins (GST fused with importin α1, α3, α5, α7, β1, or GST alone and pulled down with glutathione sepharose beads GE Healthcare). **(B)** *In vitro* interaction of pV with transportin-SR (TRN-SR). *In vitro* synthesized and [^35^S]-labeled BAdV-3 pV was incubated with purified GST fusion proteins (GST fused with TRN-SR2 or GST alone and pulled down with glutathione sepharose beads GE Healthcare). **(C)** Schematic representation of pV mutants. The thick black line represents BAdV-3 pV gene. Thin broken line represents the deleted regions. The numbers above represent amino acids of BAdV-3 pV. The name of the plasmid is depicted on the right. **(D)** *In vitro* interaction of pV mutants with importin α-3. *In vitro* synthesized and [^35^S]-labeled BAdV-3 pV mutants were incubated with purified GST-α-3 or GST alone and pulled down with glutathione sepharose beads. Samples from **(A,C)** were separated by 10% SDS-PAGE and exposed to a phosphor screen. The exposed phosphor screen was visualized by Molecular Imager FX (Bio-Rad). 5% of the input radio-labeled pV mutants were used as control.

Members of the importin super family play an important role in the nuclear transport of proteins. Since the transport of some adenovirus proteins requires importins (Kohler et al., 1999; Wodrich et al., 2006; Paterson et al., 2012; Ayalew et al., 2014; Kulshreshtha et al., 2014; Said et al., 2018; Woldemariam et al., 2020), we performed a GST (glutathione-S-transferase) pull down assay using purified GST-fusion proteins of importin α-1, importin α-3, importin α-5, importin α-7, or importin β-1 individually immobilized on glutathione-sepharose beads with radio-labeled *in vitro* synthesized BAdV-3 pV. As seen in Figure 5A, GST-importin α3 was able to bind pV (lane 5) as a similar protein was observed in input protein control (lane 1). No radio-labeled pV was observed when purified GST alone (lane 7) or GST fusions of importin β1 (lane 2), importin α7 (lane 3), importin α5 (lane 4), or importin α1 (lane 6) bound to glutathione-sepharose beads were used in pull down assays. These results suggested that pV utilizes importin α3 a member of the importin α/β pathway for nuclear localization.

Recently, we demonstrated that BAdV-3 33K interacts with transportin-3 (Kulshreshtha et al., 2014). Transportin-3 (transportin SR) is a novel member of the importin β/transportin family, which binds to RS (argine, serine)-rich domains of SR (serine/arginine) proteins and helps in the transport of these proteins to the nucleus (Hindley et al., 2007; Kulshreshtha et al., 2014). To determine if pV binds to transportin -3 (TRN- 3), GST pull down assay was performed using GST alone or GST-transportin fusion protein and *in vitro* [^35^S] methionine-labeled pV. As seen in Figure 5B, a protein could be observed in input protein control (lane 1). However, no similar protein could be detected bound to GST-TRN-3 (transportin-3) fusion protein (Lane 2) or GST alone (lane 3). These results suggest that BAdV-3 pV does not interact with and utilize transportin-3 for nuclear localization.

Like pV (Figures 5C,D), GST-importin α3 bound to glutathione-sepharose was able to bind radiolabeled pV.d18 (deletion of amino acids 190–210 and 380–423), pV.d19 (deletion of amino acids 81–120 and 190–210), and pV.d17 (deletion of amino acids 81–120 and 380–423), albeit with less intensity. However, no such interaction was observed when GST-importin α-3 fusion bound to glutathione-sepharose was used to pull down pV.d15 containing deletions of amino acids 81–120, 190–210, and 380–423.

### Construction of BAdV-3s Expressing Mutant pV Proteins

**FIGURE 6:**
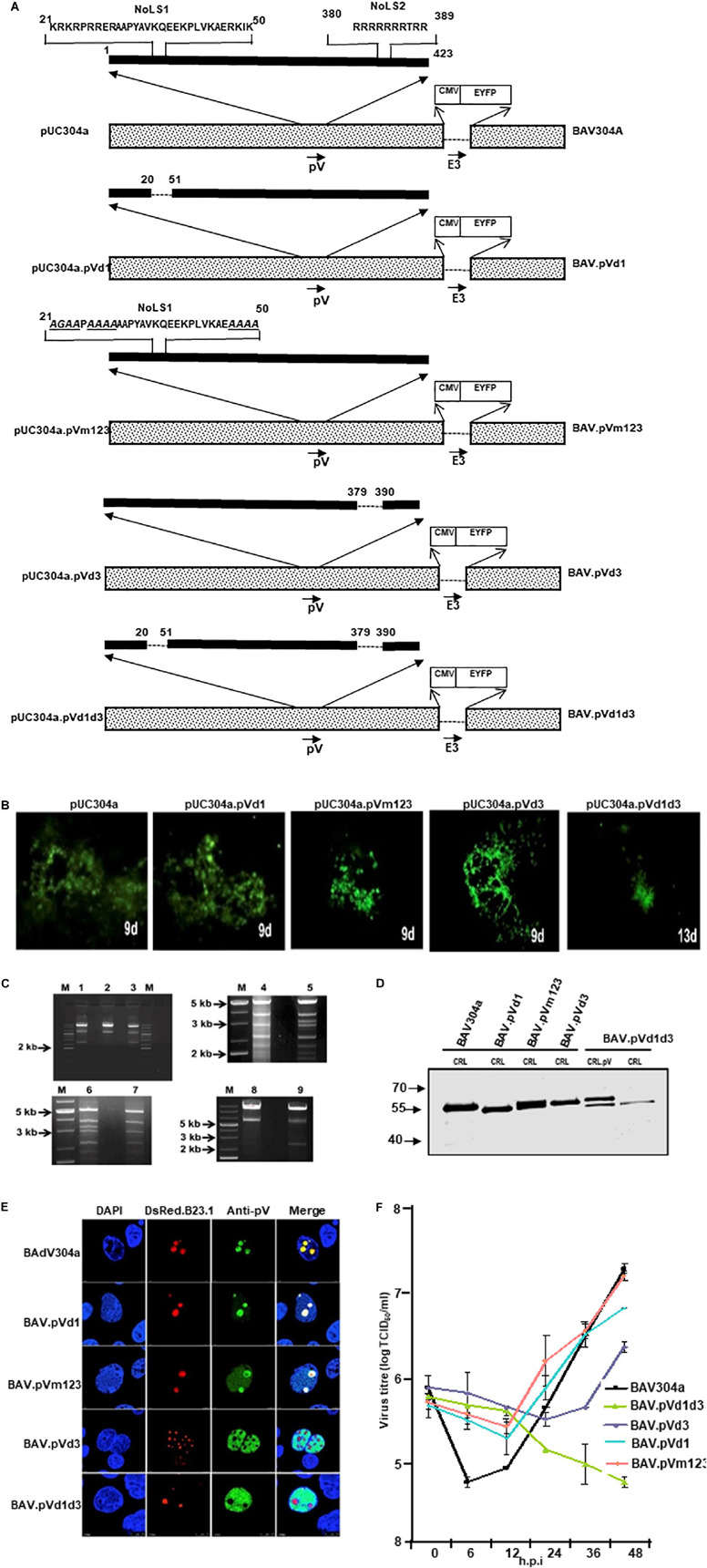
L2 pV. **(A)** Schematic representation of BAdV-3 Genomes. Dotted box represents BAdV-3 genome, and thick black line represents pV sequence. The thin line depicts the deleted regions. The arrows represent the direction of transcription. The amino acid numbers of pV are shown. The substituted amino acids (alanines\glycines) of NoLS1 are underlined and shown in italics. E3 (early region 3); nucleolar localization signal (NoLS1, NoLS2); CMV (human cytomegalovirus immediate early promoter); EYFP (enhanced yellow fluorescent protein). The name of the plasmid is depicted on the left. The name of the virus is depicted on the right. **(B)** Fluorescent microscopy. The VIDO DT1 or CRL.pV cells transfected with indicated plasmid DNAs were observed for the appearance of fluorescent cells and cytopathic effects. The numbers represent the day the observation was made after transfection. **(C)** Restriction enzyme analysis of recombinant BAdV-3 genome. The DNAs were extracted from MDBK or CRL.pV cells infected with BAV304a (lanes 2, 5, 6, and 8), BAV.pVd1 (lane 1), BAV.pVm123 (lane 3), BAV.pVd3 (lane 4), and BAV.pVd1d3 (lanes 7 and 9) as described previously (23), digested with *Xba*I (lanes 1, 2, 3, 8, and 9) or *Pst*1 (lanes 4, 5, 6, and 7) and analyzed by agarose gel electrophoresis. **(D)** Western Blot. Proteins from lysates of CRL or CRL.pV cells infected with BAV304a, BAV.pVd1, BAV.pVm123, BAV.pVd3, and BAV.pVd1d3 were separated by 10% SDS-PAGE, transferred to nitrocellulose membrane and probed by Western blot using anti-pV serum. The membrane was visualized by Odyssey^®^ CLx Imaging System (LI-COR). **(E)** Confocal microscopy. CRL cells were transfected with plasmid pDsRed.B23.1 DNA and infected with indicated mutant BAdV-3s (depicted on the left). Infected cells were fixed at 48 h post-infection. The DsRed.B23.1 was visualized by direct fluorescence microscopy. BAdV-3 pV was visualized by indirect immunofluorescence microscopy using anti-pV serum and Alexa Fluor 647-conjugated goat anti-rabbit IgG. The nuclei were stained with DAPI. **(F)** Virus titer. Monolayers of MDBK cells were infected with BAV304a or recombinant BAdV-3s. At different time points post-infection, the cells were freeze-thawed and titrated on CRL.pV cells as described. Values represent averages of two independent repeats and error bars indicate the standard deviations.

To determine if the potential NoLSs are required for efficient replication of BAdV-3, we constructed full length plasmid genomic clones expressing mutant pV containing deletion of potential NoLSs and\or substitutions of basic residues with alanine\glycine of potential NoLS1 (Figure 6A). A monolayer of VIDO DT1 (31: cotton rat lung cells expressing I-Sce1 endonuclease) cells were transfected with 5–7.5 μg of individual plasmid DNAs. The cytopathic effects appeared between 9–15 days (Figure 6B). However, repeated transfection of VIDO DT1 (Du and Tikoo, 2010) cells with plasmid pUC304a.pVd1d3 did not produce any cytopathic effects. Moreover, reinfection of fresh VIDO DT1 (Du and Tikoo, 2010) with supernatants of infected cell lysates containing mutant viruses (Figure 6A) named BAV.pVd1 (deletion of amino acid 21–50), BAV.pVm123 (containing substitutions of basic residues of all three motifs of amino acids 21–50), and BAV.pVd3 (containing deletion of amino acids 380–389) produced infectious virions. In contrast, reinfection of fresh VIDO DT1 (Du and Tikoo, 2010) with supernatant of cell lysates potentially containing mutant BAV.pVd1d3 (containing deletion of amino acid 21–50 and amino acid 380–389) did not produce any infectious virion (Data not shown), suggesting that deletion of NoLS1 (amino acids 21–50) and NoLS2 (amino acid 380–389) is lethal for the production of viable progeny virions.

To produce mutant BAV.pVd1d3, CRL.pV cells (22: CRL cells expressing BAdV-3 pV) were transfected with 5–7.5 μg of *Pac*I digested plasmid pUC304a.pVd1d3 DNA. The cytopathic effects were observed after 13 days (Figure 6B). To purify the mutant viruses, the MDBK cells infected with BAV.pVd1, BAV.pVm123, or BAV.pVd3 (Figure 6A), or CRL.pV cells (Zhao and Tikoo, 2016) infected with BAV.pVd1d3 were collected, freeze-thawed, and purified by CsCl density gradient purification.

The presence of the desired mutations was confirmed by DNA sequencing and restriction enzyme digestion of virion DNAs. Since an additional *Xba*I recognition site was introduced into mutant BAV.pVd1 or BAV.pVm123 genomes, the viral genomes were digested with *Xba*I. As seen in Figure 6C, BAV.pVd1 (Lane 1) and BAV.pVm123 (lane 3) genomes had a band of 2.4 kb, which was missing in BAV304a (lane 2). Similar analysis of *Xba*I digested BAVd1d3 (lane 9) genome detected an expected band of 2.4 kb, but not in BAV304a (lane 8). Since an additional *Pst*I recognition site was introduced to the viral genome BAV.pVd3, the viral genome was digested with *Pst*I. As seen in Figure 6C, a 3.2 kb band was detected in BAV304a (lane 5) but not in BAV.pVd3 (lane 4). Similar analysis of *Pst*I digested BAV.pVd1d3 genome detected an expected band of 3.2 kb in BAV304a (lane 6) but not in BAV.pVd1d3 (lane 7).

The ability of the mutant BAdV-3s to express pV protein was analyzed by Western blot analysis of proteins from the lysates of virus-infected cells. As seen in Figure 6D, protein bands of expected molecular weights could be detected in the lysates of CRL cells (Papp et al., 1997) infected with mutant BAV.pVd1, BAV.pVm123, BAV.pVd3, or BAV.pVd1d3. The ability of mutant BAV.pVd1d3 to express pV protein was analyzed in both CRL (Papp et al., 1997) and CRL.pV (22: CRL expressing BAdV-3 pV) cells. As expected, two proteins of 55 kDa (representing wild-type pV expressed in CRL.pV cells; 30) and 53 kDa (representing mutant pV expressed in BAV.pVd1d3) were detected in lysates of CRL.pV cells (Zhao and Tikoo, 2016) infected with BAV.pVd1d3, while only the 53 kDa band was detected in CRL cells (Papp et al., 1997) infected with BAV.pVd1d3.

### Sub Cellular Localization of Mutant pV Protein in Recombinant BAdV-3 Infected Cells

To determine the effect of deletions or amino acid substitutions on nucleolar localization of pV, CRL cells (Papp et al., 1997) were transfected with plasmid pDsRed.B23.1 DNA. At 48 h post transfection, the cells were infected with BAV304a or individual mutant BAdV-3s. At 24 h post infection, the cells were analyzed by immunofluorescence using anti-pV sera. As seen in Figure 6E, pV localized mainly in the nucleoli of BAV304a or BAV.pVd1 infected cells. Similarly, pV localized predominantly in the nucleoli of BAV.pVm123 infected cells. As expected, pV localized in the nucleus of BAV.pVd1d3 infected cells. In contrast, pV localized to the nucleus in BAV.d3 infected cells. These results suggested that subcellular localization of mutant pV expressed in transfected cells and BAV.d3 infected cells appears differently.

### Growth Kinetics of Viruses

To examine if the deletion\mutation of pV NoLs affects BAdV-3 replication, we compared the ability of the mutant viruses and BAV304a to grow in MDBK cells (ATCC CCL22), The virus infected cells were harvested at indicated time points post infection, freeze-thawed 3–5 times, and then cell lysates were used to determine the virus titers by TCID_50_ assay. As seen in Figure 6F, after 48 h, virus titer of mutant BAVpVd1, BAV.pVm123, and BAV.pVd3 appeared 0.6 to 1.0 log less compared to BAV304a. In contrast, mutant BAV.pVd1d3 did not replicate in MDBK cells.

### Analysis of Gene Expression in Mutant Virus-Infected Cells

**FIGURE 7:**
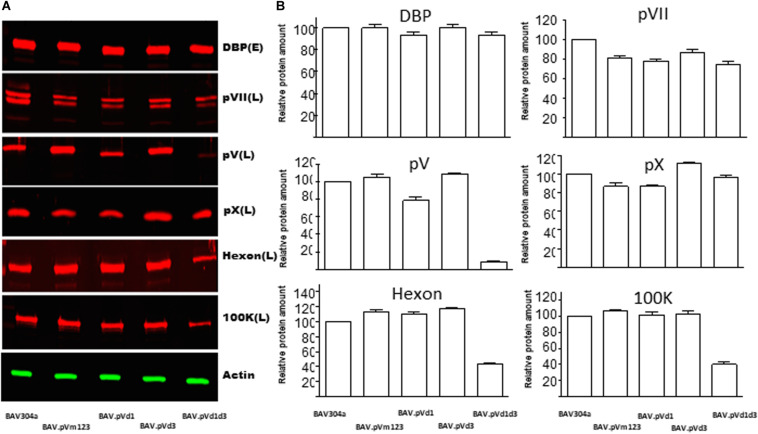
Analysis of gene expression in mutant BAdV-3 infected cells. **(A)** Proteins from lysates of CRL cells infected with indicated mutant BAdV-3s were separated by 10% SDS-PAGE, transferred to nitrocellulose, and probed with protein specific antisera and Alexa Fluor 680 conjugated goat anti-rabbit antibody (Invitrogen). β-actin was used as a loading control and was detected using anti-β-actin monoclonal antibody (Sigma-Aldrich) and IRDye800 Conjugated goat anti-mouse antibody (Rockland). Protein names are depicted on the right of the panel. E (Early), L(Late), and DBP (DNA binding protein). **(B)** The values were analyzed by using Odyssey^®^ CLx Imaging System (LI-COR). Values represent averages of two independent repeats and error bars indicate the standard deviations.

Since the deletion\mutation of pV NoLS influences the viral growth kinetics, we investigated the effects of pV NoLS deletion/mutations on the expression of early and late proteins in mutant BAdV-3-infected cells by Western blot using protein specific antisera. As seen in Figure 7A, anti-DBP serum (Zhou et al., 2001), anti-pVII serum (Paterson, 2010), anti-pV serum (this study), anti-pX serum (Paterson, 2010), anti-hexon serum (Kulshreshtha et al., 2004), and anti-100K serum (Makadiya et al., 2015) detected proteins of expected molecular weights both in BAV304a and mutant virus-infected cells. Densitometer analysis of protein production (Figure 7B) showed no significant differences in the expression of DBP or pX in any mutant BAdV-3 infected cells compared to BAV304a infected cells. Similarly, there was no dramatic change in the expression of pVII in mutant infected cells compared to BAV304a infected cells. However, the amount of hexon and 100K was significantly reduced in BAV.pVd1d3 infected cells compared to BAV304a infected cells. Moreover, the expression of pV was severely reduced in mutant BAVd1d3 infected cells compared to BAV304a, BAV.pVd1, BAV.pVm123, and BAV.pVd3 infected cells.

### Structural Protein Incorporation Assay

**FIGURE 8:**
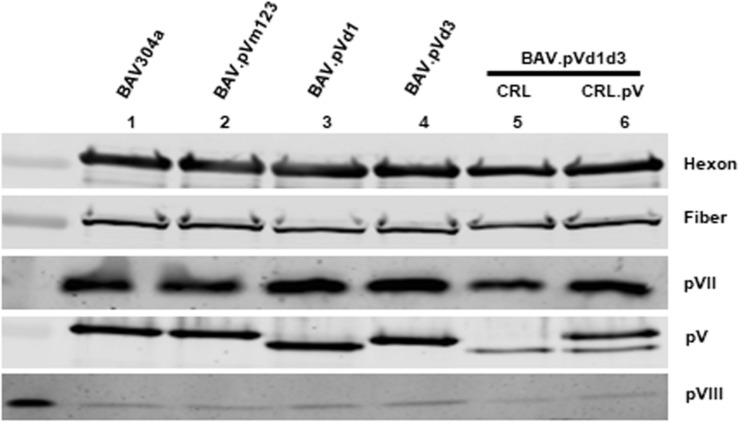
Structural protein incorporation assay. Structural proteins from the purified BAV304a (lane 1), BAV.pVd1 (lane 2), BAV.pVd1 (lane 3), BAV.pVd3 (lane4), or BAV.pVd1d3 (lane 5) grown in CRL cells and BAV.pV BAV.pVd1d3 (lane 6) grown in CRL.pV cells were separated by 10% SDS-PAGE, transferred to nitrocellulose, and probed by Western blot using protein specific antisera. Protein bands were visualized by Odyssey^®^ CLx Imaging System (LI-COR). Protein names are depicted on the right of the panel.

To determine if the decreased expression of late proteins in mutant virus infected cells influences the incorporation of structural protein in the purified virions, the proteins in the purified virus were separated by 10% SDS-PAGE, transferred to a nitrocellulose membrane, and probed with Western blot using anti-hexon (Kulshreshtha et al., 2004), anti-fiber (Wu et al., 2004), anti-pVII (Paterson, 2010), anti-pV (this study), and anti-pVIII (Ayalew et al., 2014) antisera. As shown in Figure 8, there was no detectable difference between hexon, fiber, pVII, and pVIII incorporation among recombinant BAdV-3s (Lanes 3–6) and BAV304a (Lane 1). However, the incorporation of mutant pV proteins was different. Firstly, different sized mutant pV proteins were detected in different recombinant BAdV-3s (Figure 8, lanes 3–4), indicating the deletion or mutation of pV NoLS(s). Secondly, as expected, a different pV expression pattern was observed in BAV.pVd1d3 purified from CRL (Papp et al., 1997) or CRL.pV cells (Zhao and Tikoo, 2016). As expected, two pV specific bands were detected in BAV.pVd1d3 purified from CRL.pV (Figure 8, lane 6) cells (Kulshreshtha and Tikoo, 2008), while only the lower band was detected in the BAV.pVd1d3 purified from CRL (Figure 8, lane 5) cells (Papp et al., 1997).

### Analysis of BAdV-3 Capsid Assembly

**FIGURE 9:**
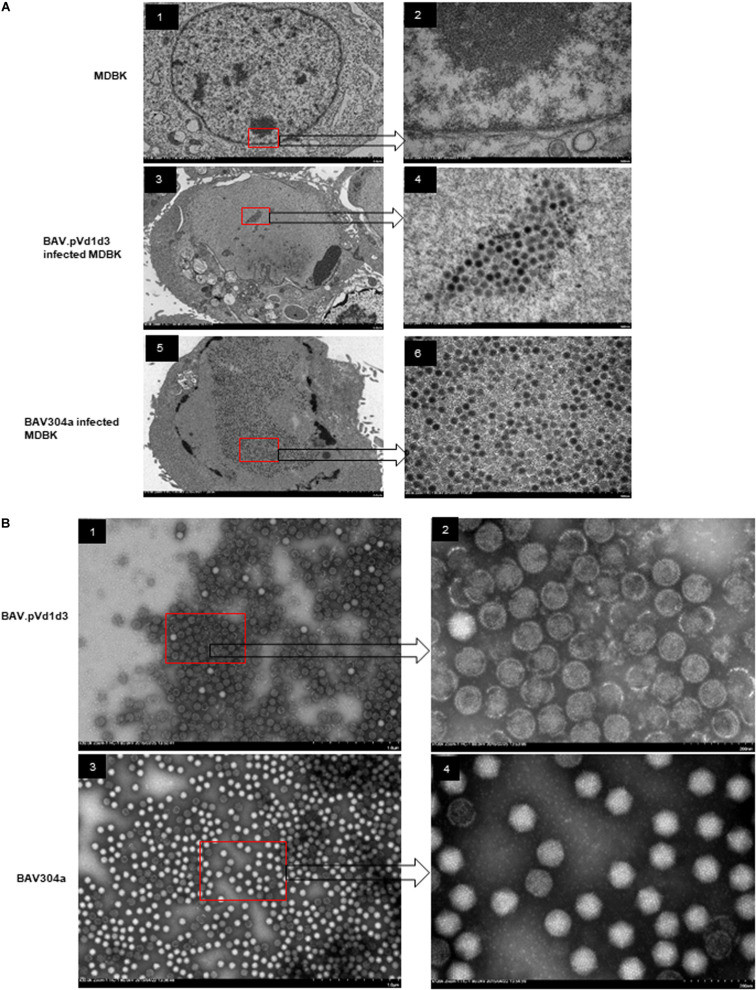
Transmission electron microscopic analysis. **(A)** Viral assembly in infected cells. Uninfected MDBK cells (panel 1, 2), MDBK cells infected with BAV.pVd1d3 (panel 3, 4) or BAV304a (panel 5, 6). The arrows depict higher magnification (60,000x) of the areas in the red boxes. **(B)** Negative staining of purified BAV304a (panel 1, 2) and BAV.pVd1d2 (panel 3, 4). The arrows depict higher magnification (120,000x) of the areas in the red boxes.

Since the expression of BAdV-3 proteins (hexon, 100K, and pV) was significantly reduced in NoLSs deleted BAdV-3 (BAV.pVd1d3), viral capsid assembly was analyzed in BAV.pVd1d3 and BAV304a infected MDBK cells by transmission electron microscopy (TEM). As seen in Figure 9A, like with BAV304a (Figure 9A, panels 5–6), capsid formation was observed in BAV.pVd1d3 infected cells (Figure 9A, panels 3–4), No such capsids were detected in uninfected cells (Figure 9A, panels 1–2).

Viral capsid assembly was also analyzed by using BAV.pVd1d3 and BAV304a viral particles purified from MDBK cells. The infected cells were harvested and freeze-thawed, and the virions were purified using CsCl gradients. As seen in Figure 9B, most purified BAV304a virions appeared icosahedral in shape with intact capsids (Figure 9B, panels 3–4). However, most purified BAV.pVd1d3 virions appeared round in shape with broken capsids (Figure 9B, panels 1–2).

### Thermostability of Recombinant BAdV-3s

**FIGURE 10:**
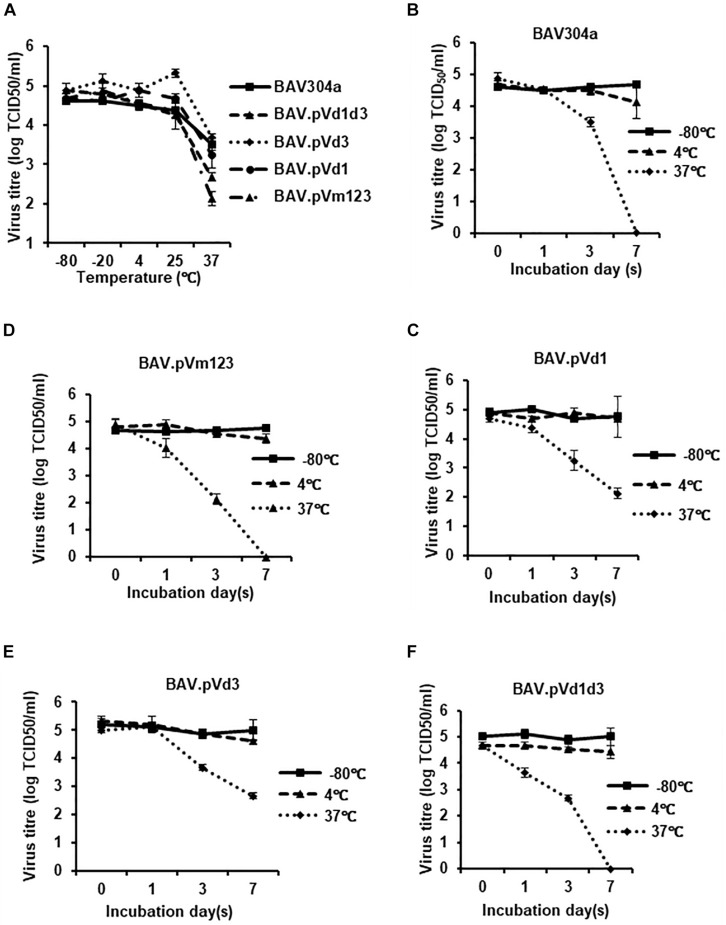
Thermostability of the recombinant BAdV-3s. **(A)** Thermostability assay of the recombinant BAdV-3s. 10^5^ TCID_50_ of BAV304a, BAV.pVm123, BAV.pVd1, or BAV.pVd3 virions purified from CRL cells or 10^5^ TCID_50_ of BAV.pVd1d3 virions purified from CRL.pV cells were incubated at −80, 20, 4, 25, or 37°C for 3 days, and the residual viral infectivity was determined with TCID_50_ on CRL.pV cells. Values represent averages of two independent repeats and error bars indicate the standard deviations. **(B–F)** 10^5^ TCID_50_ of BAV304a, BAV.pVm123, BAV.pVd1, or BAV.pVd3 virions purified from CRL cell or 10^5^ TCID_50_ of BAV.pVd1d3 virions purified from CRL.pV cells were incubated at −80, 4, or 37°C for 0, 1, 3, or 7 days. The residual viral infectivity was determined with TCID_50_ using CRL.pV cells. Values represent averages of two independent repeats and error bars indicate the standard deviations.

Deletions and mutations in viral genomes are always associated with thermo vulnerability of viral capsids (Ugai et al., 2007). To examine if the deletion or mutation of pV NoLSs leads to the decrease of BAdV-3 capsid thermostability, wild-type and mutant BAdV-3s were treated as described (Ugai et al., 2007). As seen in Figure 10A, there was no titer difference when the temperature was under 25°C. However, when viruses were incubated at 37°C, the titers dropped significantly, especially for BAV.pVd1d3 (Figure 10A) and BAV.pVm123 (Figure 10A). To assess the different dynamics of viral inactivation, wild-type and recombinant BAdV-3s were treated at −80, 4, or 37°C for 0, 1, 3, or 7 days. As seen in Figures 10B–F, after 7 days’ incubation at −80°C or 4°C, there was no detectable change in all five BAdV-3s. However, after 7 days’ incubation at 37°C, BAV304a, BAV.pVm123, and BAV.pVd1d3 lost all their infectivity, while for the single NoLS deleted recombinant viruses BAV.pVd3 and BAV.pVd1, ∼10^3^ infectious viral particles were still remaining.

## Discussion

Although adenovirus protein homologs are encoded by members of the *Mastadenovirus* genus, recent reports have demonstrated the differences in the sub cellular localization and function of homologous adenovirus proteins (Stracker et al., 2005; Blanchette et al., 2013; Cheng et al., 2013). Recently, we reported that 100K protein encoded by HAdV-5 and BAdV-3 differ in sub cellular localization and protein function (Makadiya et al., 2015). Protein V is a *Mastadenovirus* genus specific minor core protein, which localizes to both the nucleus and the nucleolus in HAdV-5 infected cells (Matthews, 2001). An earlier report suggests that transportin may be involved in the nucleolar localization of HAdV-5 pV (Hindley et al., 2007). The present study was designed to characterize BAdV-3 pV protein, identify NLSs and NoLSs, and investigate the mechanism of nuclear/nucleolar localization.

Proteins localizing to the nucleolus also localize to the nucleus and thus may contain either overlapping NLS\NoLS (Cheng et al., 2002; Sheng et al., 2004) or separate non-overlapping signals for localizing to both the nucleus and the nucleolus (Ladd and Cooper, 2004; Cros et al., 2005). The deletion analysis identified N-terminal amino acids 21–50 (NoLS1) and C-terminal amino acid 380–389 (NoLS2) as NoLS, both containing basic residues (arginine and lysine) that can function as NoLS. Both NoLS1 and NoLS2 amino acids were sufficient to direct nucleolar imports of EYFP, a non-nucleolar protein. This is consistent with an earlier report suggesting that NoLSs are rich in basic amino acids (arginine and lysine) and are predominantly localized near the N- or C-terminus of the protein (Scott et al., 2010). Deletion of a potential individual NoLS did not reduce the nucleolar localization of pV. However, deletion of both NoLSs abrogated the nucleolar localization of pV. Like NoLS1 and NoLS2, three arginine and lysine rich motifs of NoLs1 appear to have a redundant function as deletion of either NoLS or mutation of any arginine- lysine rich motif of NoLs1 did not abrogate the nucleolar localization of BAdV-3 pV. Interestingly, Vm1m2m3d3 protein appears as multi-punctuate dots in the nucleus of transected cells (Figure 3B). It is possible that NoLSs may have overlapping functions and, in the absence of NoLSs, pV shows aberrant punctuate staining in the nucleus (Tollefson et al., 2007). A similar staining pattern has been observed in stable cells expressing human cytomegalovirus IE 1 protein (Lafemina et al., 1989).

Compared to transfected cells, the deletion of NoLS2 appears to alter the nucleolar localization of mutant pV in BAdV3.pVd3 infected cells. It is possible that NoLS1 and NoLS2 function at different phases of viral replication. While NoLs1 may function at an early phase of infection, only NoLs2 may function at a late phase of infection. Similar findings have been reported for VP1 protein of porcine parvo virus (Boisvert et al., 2014).

The deletion of potential NoLS1 did not alter the nuclear localization of pV. Moreover, V.d15 (Figure 4C) containing amino acid 21–50 (NoLS1) localized predominantly in the cytoplasm of the transfected cells (Figure 4D). The fusion protein NoLS1GβGal (Figure 4E) containing amino acid 21–50 fused to GFPβGal (36; a cytoplasmic protein) showed no nuclear or nucleolar localization (Figure 4F). These results suggest that the NoLS1 does not contain the NLSs required for pV to localize to the nucleus. In contrast, mutant protein V.d16 (Figure 4C) containing amino acids 21–50 (NoLS1) and 380–389 (NoLS2) located in the nucleus and nucleolus (Figure 4D), suggesting that amino acids 380-389 can mediate V.d16 for both nuclear and nucleolar localization of pV.

The nucleolar transport usually requires binding of nucleolar constituents to protein sequences, namely NoLS, which helps to retain the protein in the nucleolus. Though there is no consensus in known NoLS sequences, NoLSs are usually rich in lysine and arginine residues, which may interact with nucleolar RNAs or other nucleolar proteins (Olson and Dundr, 2005) for their retention in the nucleolus by a charge dependent mechanism (Musinova et al., 2015). While many nucleolar viral proteins contain RNA binding motifs (Hiscox, 2007) and are retained in the nucleolus by binding to nucleolar RNAs, nucleophosmin protein contains acidic regions which bind to positively charged amino acids in putative nuclear viral proteins and retain them in the nucleolus (Adachi et al., 1993). Though NoLS1 and NoLS2 do not contain a specific amino acid sequence, both are rich in positively charged\basic residues. Since no specific NoLS sequence pattern could be defined in pV, the abundance of positively charged\basic residues appears to mediate the translocation of pV from nucleus to nucleolus, suggesting that nucleolar retention is due to electrostatic interactions. Deletion of the positively charged\basic residues (arginine and lysine) of NoLS1 and\or NoLs2 reduce the electrostatic interactions with nucleaolar contents, thus abolishing the localization of pV to the nucleolus.

Unlike nucleolar transport, nuclear import requires active transport mechanisms, which are dependent on energy, soluble factors, and functional nuclear pore complex (Valdez et al., 1994). Most of the proteins imported into the nucleus contain NLSs (Boulikas, 1993; Nigg, 1997) which interact with importin α\β and\or transportin in the cytoplasm and are transported through the nuclear pore complex into the nucleus (Kosugi et al., 2009; Kulshreshtha et al., 2014). Though bioinformatic analysis predicted amino acids 190–210 and 380–389 to act as potential NLS, deletion analysis identified three regions, including amino acid 80–120, 190–210, and 380–389, as NLS. Deletion of all three motifs is required to abolish the nuclear localization and binding of importin α-3 to each NLS motif, suggesting that each motif is functionally redundant. Separate or overlapping redundant NLSs have been identified in viral proteins including polyomavirus large T antigen (Richardson et al., 1986; Howes et al., 1996), influenza virus NS1 protein (Melen et al., 2007), adeno-associated virus 2 assembly activating protein (Earley et al., 2015), and in BAdV-3 33K (Kulshreshtha et al., 2014). It is possible that the presence of multiple BAdV-3 pV NLS with redundant functions may help promote efficient interaction with the nuclear transport system, leading to an effective nuclear transport. Support for this comes from the fact that increased binding of pV to importin α-3 could be observed in the presence of all three NLS regions (Figures 5C,D).

Earlier, overlapping karyopherin importin α-5 and transporetin-3 binding motifs were identified in the C-terminus 40 amino acid region of BAdV-3 33K protein (Kulshreshtha et al., 2014). Similarly, overlapping NLS and NoLS motifs have been identified in viral proteins (Li et al., 2011). BAdV-3 pV appears to utilize both separate NLS (amino acid 80–120 and 190–210) and NoLS (amino acid 21–25) or overlapping NLS/NoLS (amino acid 380–389) to translocate to the nucleus and nucleolus. It is likely that utilization of alternate mechanisms of nucleolar transport ensures that BAdV-3 pV performs important function(s) in the nucleus/nucleolus, necessary for the production of infectious progeny viruses.

A number of viral proteins, including HAdV-5 pVII, use multiple nuclear import pathways (Wodrich et al., 2006). Recently, we also demonstrated that nuclear import of BAdV-3 33K involves recognition of overlapping NLS motifs located in 40 amino-acid-long conserved regions of BAdV-3 33K by importin α-5 and transportin-3 (Kulshreshtha et al., 2014). Our data suggest that the nuclear import of pV appears to be mediated only by importin α-3 of the importin α/β pathway and requires amino acids 81–120, 190–210, or 380–423. Thus, the active nuclear transport of pV mediated by Imp α-3 requires at least one of the three identified NLS motifs of pV. Interestingly, the nuclear import of BAdV-3 52K (Paterson et al., 2012) and pVIII (Ayalew et al., 2014) also appears to be mediated by interacting with Imp α-3 of the classical Imp α\β dependent pathway.

Although deletion of NoLS2 affects the efficient production of progeny viruses, neither NoLS1 nor NoLS2 not appear essential for the production of viable viruses, suggesting that each NoLS motif may be functionally redundant. In contrast, deletion of both NoLS1 and NoLS2 prevented the production of viable viruses, suggesting that both NoLSs of pV are essential for the production of a viable virus. Earlier reports have demonstrated the relationship between nucleolar localization of a viral protein and viral replication (Ishida et al., 2019). Depending on the virus, nucleolar localization of a viral protein may (Ishida et al., 2019) or may not (Earley et al., 2017; Ishida et al., 2019) be essential for viral replication. Our results suggest that nucleolar localization of pV appears essential for the efficient production of infectious BAdV-3.

It is possible that nucleolar localization of pV is required for modulating the function of nucleolar protein(s) required for the efficient replication of BAdV-3. Many viruses are known to induce alterations in the nucleolus, which appear important for viral replication. Earlier reports have suggested that HAdV-5 interacts with the nucleoli of infected cells and alter the composition of nucleolar proteins (Walton et al., 1989; Lam et al., 2010). However, the nucleolar localization of only transiently overexpressed HAdV-5 pV has been shown to induce translocation of nuclephosmin 1\B23.1\NPM1 and nucleolin from the nucleolus to the cytoplasm of the transfected cells (Matthews, 2001). BAdV-3 pV colocalized with B23.1 in the nucleolus of infected (Figure 1D, panel b) or transfected (Figure 1D, panel f) cells without inducing the redistribution of B23.1 to the cytoplasm. Similarly, BAdV-3 pV interacted with nucleolin in infected cells and colocalized with nucleolin in the nucleolus of the transfected cells and did not induce the redistribution of nucleolin to the cytoplasm. In fact, interaction and co-localization of pV and nucleolin, observed in the nucleolus of BAdV-3 infected cells (Zhao, 2016), may inhibit the ability of nucleolin to interfere with adenovirus replication (Yang et al., 1994; Matthews, 2001).

Nuclear localization signals/NoLS sequences of viral proteins may also perform other functions required for efficient production of progeny virions (Earley et al., 2015). Deletion of NoLS1 and NoLS2 reduced the expression of some late viral proteins, namely hexon, 100K, and pV in infected cells, and produced thermolabile BAV.pVd1d3 virions. Although the mechanism of decreased protein production is not clear yet, the production of thermolabile virions could be due to altered protein-DNA or protein-protein interactions required for the stability of progeny virions. A previous report (Reddy and Nemerow, 2014) has suggested that the C-terminus of recombinant Ad5F5 pV can interact with pVI in the interior of adenovirus particles, and pV can also interact with pVIII C-terminus, thus forming a ternary complex to glue the peripentonal hexons (PPH), and connect them with adjacent groups of nine hexons (GON). Moreover, the N-terminus (basic amino acids rich) of pV may interact with the adenoviral genome to bridge the viral core with the capsid. It is possible that deletion of both NoLS1 (N terminal) and NoLS2 (C terminal) alters the interaction of pV with the genome and pVI, affecting efficient interaction of the core with capsid proteins and leading to the formation of fragile BAdV-3 capsids.

In conclusion, we have demonstrated that pV contain multiple non-overlapping/overlapping NLSs\NoLSs. Moreover, the NoLSs are important not only for nucleolar localization of pV but also for the production of stable infectious progeny BAdV-3 virions.

## Materials and Methods

### Cells and Viruses

Madin Darby bovine kidney (MDBK; ATCC CCL22), cotton rat lung (CRL) cells (25:, VIDO DT1) cells (31:CRL cells expressing I-SceI endonuclease), and CRL.pV (22:CRL cells expressing BAdV-3 pV) cells were cultivated in minimal essential medium (MEM; Sigma) supplemented with 10% heat-inactivated fetal bovine serum (FBS, Invitrogen). Vero cells (ATCCCCL-81) and HEK293T cells (ATCC CRL-11268) were propagated in Dulbecco’s modified eagle’s medium (DMEM) supplied with 10% FBS. BAV304a (BAdV-3 E3 region was replaced by a EYFP gene) and mutant BAdV-3s were cultivated in MDBK or CRL.pV cells.

### Antibodies

The production and characterization of antibodies raised against BAdV-3 DBP (Zhou et al., 2001), fiber (Wu et al., 2004), and 100K (Makadiya et al., 2015) have been described. Anti-hexon serum detects a protein of 98 kDa in BAdV-3 infected cells (Kulshreshtha et al., 2004). Anti-pVII serum detects two proteins of 26 and 24 kDa in BAdV-3 infected cells (Paterson, 2010). Anti-pX recognizes a protein of 25 kDa in BAdV-3 infected cells (Paterson, 2010).

To produce BAdV-3 pV specific sera, two peptides representing amino acid 1–24 (XZ1) and amino acids 180–212 (XZ2) were synthesized by Genscript. Rabbits were immunized with individual (500 μg/rabbit) peptide conjugated to keyhole limpet haemocyanin emulsified with Freund’s complete adjuvant (Sigma) followed by two injections of ovalbumin conjugated individual peptide (300 μg/rabbit) in Freund’s incomplete adjuvant (Sigma), 3 weeks apart. Sera were collected 10 days after the third injection and tested for specificity by Western blotting. Anti-RPA194 antibody (C-1; Santa Cruz Biotechnology),Anti-β-actin monoclonal antibody (Sigma-Aldrich), Alexa Fluor 488-conjugated goat anti-rabbit IgG (Jackson Immunoresearch), TRITC-conjugated goat anti-mouse IgG (Jackson Immunoresearch), TRITC-conjugated goat anti-rabbit IgG (Jackson Immunoresearch), Alexa Fluor 647-conjugated goat anti-rabbit IgG (Invitrogen), Alexa Fluor 680 conjugated goat anti-rabbit antibody (Invitrogen), and IRDye800 conjugated goat anti-mouse antibody (Rockland) were purchased.

### Construction of Plasmids

The plasmids used in this study were constructed using standard DNA manipulation techniques (Sambrook and Russell, 2000) and are described elsewhere (Supplementary File 1).

### Isolation of pV Nucleolar Localization Signal Deleted BAdV-3 Mutants

To isolate mutant BAdV-3s, we constructed full-length BAdV-3 plasmids containing mutant BAdV-3 genomic DNAs as described (Chartier et al., 1996).

#### Plasmid pUC304a.pVd1

A 972 bp DNA fragment was amplified by PCR using primers M-F and d(21–50)-F1-R (Table 1), with plasmid pcV DNA as a template. Similarly, an 1134-bp DNA fragment was amplified by PCR using primers d(21–50) F2-F and pV-*Xho*I-R (Table 1), and plasmid pcV DNA as a template. In the third PCR, these two PCR fragments were annealed and used as a DNA template to amplify a 2068-bp DNA fragment by overlapping PCR using primers M-F and pV-*Xho*I-R (Table 1). A 1171-bp *Eco*RI-*Xho*I DNA fragment of the final PCR product (2068 bp) was isolated and ligated to *Eco*RI-*Xho*I digested plasmid pcDNA3 to create plasmid pcV.d1. A 528-bp *Eco*RI*-Nhe*I DNA fragment of plasmid pcV.d1 was isolated and ligated to *Eco*RI*-Nhe*I digested pMCS.pV to create plasmid pMCS.pVd1.

**TABLE 1:** List of primers used for PCR.

Name of the primer	Primer sequence
M-F	5′- TCTGCTCTGA TGCCGCATAGTTAAGCC-3′
d(21–50)-F1-R	5′- CGCTTTCTAGAGCCGCGGTAAATCTCAGGCGCCAC GATGT C-3′
d(21–50)-F2-F	5′-TCGTGGCGCCTGAGATTTACCGCGGCTCTAGAAAGCGGG CCTTG-3′
pV-*Xho*I-R	5′-AATACTCGAGAGCGCTTAACGGCGGAGCCGGGTTAC-3′
M12-F1-R	5′-CTGCAGCAGCTGCTGCGGGTGCAGCTCCTGCGTAAA TCTC AGGCGCCACGATG-3′
M12-F2-F	5′-CGCAGGAGCTGCACCCGCAGCAGCTGCTGCAGCA CCGTA TGCTGTGAAG-3′
M3-F1-R	5′-TTTCTAGAGCCGCGAGCAGCTGCTGCCTCCGCCT TTACTA AAGGCTTCTC-3′
M3-F2-F	5′-TTAGTAAAGGCGGAGGCAGCAGCTGCTCGCGGC TCTAG AAAGCG GGCCTTG-3′
pV-*Eco*RI-F	5′-GGAGCC GAATTCATGGCCTCCTCTCGGTTGATTA AAGAAG-3′
pV-d(380–389) F1-R	5′-CAGCGCTGAGGCGGGGAGTCGCGACTGCAGGCAGGC GCACA C-3′
pV-d(380–389) F2-F	5′-GTGTGCGCCTGCCTGCAGTCGCGACTCCCCGCC TCAGCG CTG-3′
dV-F2-R	5′-GTCC-ATGGCGTGTTAACAAGCTGTG-3′
M-F	5′- TCTGCTCTGA TGCCGCATAGTTAAGCC-3
d(21–50)-F1-R	5′- CGCTTTCTAGAGCCGCGGTAAAT CTCAGGCGCCACGA TGTC –3′
d(21–50)-F2-F	5-TCGTGGCGCCTGAGATTTACCGCGGCTCTAGAAAGCGGG CCTTG-3′
pV-*Xho*I-R	5′-AATAC TCGAGAGC G CTTAACGGCGGAGCCGGGTTA C-3′
M12-F1-R	5′-CTGCAGCAGCTGCTGCGGGTGCAGCTCCTGCGTAAAT CTC AGGCGCCACGATG-3′
M12-F2-F	5′-CGCAGGAGCTGCACCCGCAGCAGCTGCTGCAGCAC CGTA TGCTGTGAAG-3′
M3-F1-R:	5′-TTTCTAGAGCCGCGAGCAGCTGCTGCCTCCGCCTTTA CTA AAGGCTTCTC-3′
M3-F2-F	5-TTAGTAAAGGCGGAGGCAGCAGCTGCTCGCGGCTCTAG AAAGCG GGCCTTG-3′
pV-*Eco*RI-F	5′- GGAGCC GAATTCATGGCCTCCTCTCGGTTGATTAAAGAA G-3′
pV-d(380–389) F1-R	5′-CAGCGCTGAGGCGGGGAGTCGCGACTGCAGGCAGGC GCACAC-3′
pV-d(380–389) F2-F	5′-GTGTGCGCCTGCCTGCAGTCGCGACTCCCCGCC TCAGCGCTG-3′
dV-F2-R	5′-GTCC-ATGGCGTGTTAACAAGCTGTG-3-

Finally, a 6.2-kb *Eco*RV*-Bst*1107I fragment of plasmid pMCS.pVd1 was isolated and recombined with *Sbf*I digested plasmid pUC304a.dV DNA in *Escherichia coli* BJ5183 (Chartier et al., 1996) to generate plasmid pUC304a.pVd1.

#### Plasmid pUC304a.pVm123

A 986-bp DNA fragment was amplified by PCR using primers M-F and M12-F1-R (Table 1) and plasmid pcV DNA as a template. Similarly, a 1025-bp DNA fragment was amplified by PCR using primers M12-F2-F and pV-*Xho*I-R (Table 1), and plasmid pcV DNA as a template. In the third PCR, two fragments were annealed and used to amplify a 2159-bp DNA fragment by overlapping PCR using primers M-F and pV-*Xho*I-R (Table 1). Finally, a 1261-bp *Eco*RI-*Xho*I DNA fragment of the PCR product (2159 bp) was isolated and ligated to *Eco*RI-*Xho*I digested plasmid pcDNA3 to generate plasmid pcDNA3-pV-m12.

To create pcV.m123, a 1059-bp DNA fragment was amplified by PCR using primers M-F and M3-F1-R (Table 1), and plasmid pcDNA3-pV-m12 as a template. An 1141-bp DNA fragment was amplified by PCR using primers M3-F2-F and pV-*Xho*I-R (Table 1), and plasmid pcDNA3-pV-M12 DNA as a template. In the third PCR, these two DNA fragments were annealed and used to amplify a 2159-bp DNA fragment by overlapping PCR using primers M-F and pV-*Xho*I-R (Table 1). Finally, a 1261-bp DNA fragment of the PCR product (2159-bp) was isolated and ligated to *Eco*RI-*Xho*I digested plasmid pcDNA3 to generate plasmid pcV.m123.

A 618-bp *Eco*RI*-Nhe*I fragment of plasmid pcV.m123 was isolated and ligated to *Eco*RI*-Nhe*I digested plasmid pMCS.pV to create plasmid pMCS.pVm123. The *Sbf*I digested plasmid pUC304-dV was recombined with a 6.3-kb *Eco*RV*-Bst*1107I DNA fragment of plasmid pMCS.pVm123 in *E. coli* BJ5183 (Chartier et al., 1996), creating plasmid pUC304a.pVm123.

#### Plasmid pUC304a.pVd3 and pUC304a.pVd1d3

An 1171-bp fragment was amplified by PCR using primers pV-*Eco*RI-F and F1-R (Table 1), with plasmid pMCS.pV DNA as a template. Similarly, a 661-bp fragment was amplified by PCR using primers pV-d (380–389) F2-F and dV-F2-R (Table 1), and plasmid pMCS.pV DNA as the template. In the third PCR, two PCR fragments were annealed and used to amplify a 1790-bp DNA fragment by overlapping PCR using primers pV-*Eco*RI-F and dV-F2-R (Table 1). Finally, a 650-bp *Sac*I*-Hpa*I fragment of PCR product (1790 bp) was isolated and ligated to *Sac*I*-Hpa*I digested plasmid pMCS.pV and pMCS.pVd1 to create plasmid pMCS.pVd3 and pMCS.pVd1d3, respectively.

The *Sbf*I digested plasmid pUC304a.dV was recombined with a 6.2-kb *Eco*RV*-Bst*1107I fragment of plasmid pMCS.pVd3 or plasmid pMCS.pVd1d3 in *E. coli* BJ5183 (Chartier et al., 1996) to generate plasmid pUC304a.pVd3 and plasmid pUC304a.pVd1d3, respectively.

### Western Blotting

Proteins in purified recombinant BAdV-3s or infected cells were separated by 10% SDS-PAGE, transferred to nitrocellulose membrane, and probed by Western blot using protein specific antiserum and alkaline phosphatase (AP)-conjugated goat anti-rabbit IgG (Sigma) or Alexa Fluor 680 conjugated goat anti-rabbit antibody (Invitrogen).

### Immunofluorescence Microscopy

Cells infected with BAV304a, mutant BAdV-3s, or transfected with plasmid DNAs were processed as described earlier (Wodrich et al., 2006). Finally, cells were mounted by mounting buffer (Vector Laboratories Inc.) with DAPI and imaged under confocal microscope TCS SP5 (Leica).

### GST-Pull Down Assay

The GST pull down assay was performed as described (Kulshreshtha et al., 2014) using [^35^S] Methionine-labeled pV proteins.

### Isolation of Mutant BAdV-3s

Monolayers of VIDO DT1 (31; CRL cells expressing I-SceI recombinase) cells or CRL.pV (22; CRL cells expressing BAdV-3 pV) cells in six-well plates were transfected with 5–7.5 μg of individual plasmid DNA with Lipofectamine 2000 (Invitrogen). At 4 h post-transfection, the media was replaced with fresh MEM containing 2% FBS. Transfected cells showing cytopathic effect (CPE) were harvested, freeze-thawed three times, and propagated in MDBK cells.

### CsCl Gradient Centrifugation

Monolayers of CRL (Papp et al., 1997) or CRL.pV (22;CRL cells expressing BAdV-3 pV) cells in T-150 Flasks were infected with wild type or mutant BAdV-3s at a multiplicity of infection of 5. At 48 h post-infection, the cells were collected and resuspended in 5 ml medium. After freeze thawing three times, the cell lysates were subjected to CsCl density gradient centrifugation at 35,000 rpm for 1 h at 4°C. The bands containing viruses were collected and subjected to a second centrifugation at 35,000 rpm for 16 h at 4°C. Finally, the virus band was collected, dialyzed three times to remove trace amounts of cesium chloride, and stored in small aliquots at -80°C.

### Virus Single Cycle Growth Curve

Monolayers of cells in 24-well plates were infected with wild type or mutant BAdV-3s at a MOI of 1. At indicated time points post-infection, the infected cells were harvested, lysed by freeze-thawing three times to release the virus into medium, and then used to determine virus titers by TCID_50_ in CRL.pV cells (Zhao and Tikoo, 2016).

### Protein Expression Analysis

Monolayers of cells in six-well plates were infected with BAV304a or mutant BAdV-3s at an MOI of 1. At 24 h post-infection, infected cells were harvested and probed by Western blot using protein-specific rabbit antisera and mouse anti-β-actin as primary antibodies (Sigma), Alexa Fluor 680 goat anti-rabbit (Invitrogen), and IRDye 800 goat anti-mouse (Rockland), respectively, as secondary antibodies. At last, the membranes were imaged and analyzed by using the Odyssey^®^ CLx Imaging System (LI-COR).

### Virus Thermostability Assay

To determine the thermostability of BAV304a and mutant BAdV-3s, 10^5^ purified infectious viral particles were incubated at different temperatures (−80, −20, 4, 25, and 37°C) for 3 days in PBS containing 10% glycerol. To assess the different dynamics of viral inactivation, 10^5^ infectious purified viral particles were incubated at different temperatures (−80, 4, and 37°C) for 0, 1, 3, and 7 days in PBS containing 10% glycerol. Finally, TCID50 was used to titrate the remaining infectivity.

### Transmission Electron Microscopy

Monolayers of cells were infected with BAV304a or BAV.pVd1d3 at an MOI of 5. At 48 h post-infection, the cells were harvested. The virus infected cells of CsCl-purified BAdV-3 virions were processed as described earlier (Zhao and Tikoo, 2016; Gaba et al., 2017).

## Data Availability Statement

Some data presented in the study are included in the article/Supplementary Materials, further inquiries can bedirected to the corresponding author/s.

## Ethics Statement

The animal study was reviewed and approved by Animal Research Ethics Board, Uni of Saskatchewan.

## Author Contributions

ST and XZ conceived and designed the experiments. XZ performed the experiments. ST and XZ analyzed the data and wrote the manuscript. Both authors contributed to the article and approved the submitted version.

## Conflict of Interest

The authors declare that the research was conducted in the absence of any commercial or financial relationships that could be construed as a potential conflict of interest.
