# Supplementary material for: Nuclear and Nucleolar Localization of Bovine Adenovirus-3 Protein V
Source: Front Microbiol. 2021 Jan 6;11:579593. doi: 10.3389/fmicb.2020.579593 (PMC7815533; doi:10.3389/fmicb.2020.579593)
Supplement: Supplementary file 1 [file Data_Sheet_1.PDF]

Supplementary File 1

Plasmid pDsRed.B23.1 was a gift from Dr Denis Archambault University of Quebec, Canada. Plasmids encoding GST alone or fused with importin-α1, importin-α3, importin-α5, importin-α7 or importin-β-1 were gifts from Dr. M. Köhler and have been earlier described (Depping et al., 2008). Plasmid pGST-TRN-SR2 plasmid was a gift from Dr. Woan-Yuh Tarn and has been described before (Lai et al., 2001). Plasmid pUC304a contains the BAV304a genome flanked by *I-SceI* endonuclease recognition sites (Du and Tikoo, 2010). Plasmid pCMVGbeta expresses a fusion protein of GFP/β-galactosidase and has been described (Wu et al., 2004).

a) Plasmid pcV A 1292-bp DNA fragment was amplified by PCR using primers pV-EcoRI-F and pV-XhoI-R (Table A1), and pUC304A+ as a DNA template. A 1272-bp *EcoRI-XhoI* fragment of the 1292-bp DNA fragment was ligated to a 5413-bp *EcoRI-XhoI* DNA fragment of plasmid pcDNA3 to create pcV.

b) Plasmid pcV.d2.A 599-bp DNA fragment was amplified by PCR using primers pV-EcoRI-F and d(190-210)-F1-R (Table A1), and plasmid pcV as a template. A 657-bp DNA fragment was amplified by PCR using primers d(190-210)-F2-F and pV-XhoI-R (Table A1), and plasmid pcV as a template. In the third PCR, two PCR fragments were annealed and used to amplify a 1299-bp DNA fragment by overlapping PCR using primers pV-EcoRI-F and pV-XhoI-R. A 1225-bp *Eco*RI-*Xho*I DNA fragment of the 1299-bp fragment was ligated to *Eco*RI-*Xho*I digested plasmid pcDNA3 to create plasmid pcV.d2.

c) Plasmid pcV.d3.A 1171-bp fragment was amplified by PCR using primers pV-EcoRI-F and d(380-389) F1-R (Table A1), and plasmid pcV as a template. A 592-bp fragment was amplified

by PCR using primers d(380-389) F2-F and d(380-389)-F2-R (Table A1), and plasmid pcV as a template. In the third PCR, two PCR fragments were annealed and used to amplify a 1713-bp DNA fragment by overlapping PCR using primers EcoRI-F and d(380-389)-F2-R. A 1247-bp *EcoRI-XhoI* fragment of 1713 bp fragment was ligated to *Eco*RI-*Xho*I digested plasmid pcDNA3 to create plasmid pcV.d3.

d) Plasmid pcV.d1d2.A 761-bp *Sna*BI band of pcV.d1 was isolated and ligated to *Sna*BI digested pcVd2 to create pcV.d1d2.

e) Plasmid pcV.d1d3, pcV.d2d3 and pcV.d1d2d3.A 552-bp *Age*I-*Xho*I fragment of pcV.d3 was isolated and ligated to *Age*I-*Xho*I digested pcV.d1, pcV.d2 and pcV.d1d2 to construct pcV.d1d3, pcV.d2d3 and pcV.d1d2d3, respectively.

f) Plasmid pcV.m1d3.A 984-bp DNA fragment was amplified by PCR using primers M-F and m1-F1-R (Table A1), and plasmid pcV.d3 as a template. An 1102-bp DNA fragment was amplified by PCR using primers m1-F2-F and pV-XhoI-R (Table A1), and plasmid pcV.d3 as a template. In the third PCR, two PCR fragments were annealed and used to amplify a 2043-bp DNA fragment by overlapping PCR using primers M-F and pV-XhoI-R. An 1144-bp *Eco*RI-*Xho*I DNA fragment of the 2043-bp fragment was ligated to *Eco*RI-*Xho*I digested plasmid pcDNA3 to create plasmid pcV.m1d3

g) Plasmid pcV.m2d3.A 996-bp DNA fragment was amplified by PCR using primers M-F and m2-F1-R (Table A1), and plasmid pcV.d3 as a template. A 1087-bp DNA fragment was amplified by PCR using primers m2-F2-F and pV-XhoI-R (Table A1), and plasmid pcV.d3 as a template. In the third PCR, two PCR fragments were annealed and used to amplify a 2043-bp DNA fragment by overlapping PCR using primers pV-M-F and pV-XhoI-R. An 1144-bp *Eco*RI-

*Xho*I DNA fragment of the 2043-bp fragment was ligated to *Eco*RI-*Xho*I digested plasmid pcDNA3 to create plasmid pcV.m2d3.

h) Plasmid pcV.m12d3.A 987-bp DNA fragment was amplified by PCR using primers M-F and m12-F1-R (Table A1), and plasmid pcV.d3 as a template. A 1088-bp DNA fragment was amplified by PCR using primers m12-F2-F and pV-XhoI-R (Table A1), and plasmid pcV.d3 as a template. In the third PCR, two PCR fragments were annealed and used to amplify a 2043-bp DNA fragment by overlapping PCR using primers pV-M-F and pV-XhoI-R. An 1144-bp *Eco*RI-*Xho*I DNA fragment of the 2043-bp fragment was ligated to *Eco*RI-*Xho*I digested plasmid pcDNA3 to create plasmid pcV.m12d3.

i) Plasmid pcV.m3d3, pcV.m13d3, pcV.m23d3and pcV.m123d3.Four 1060-bp DNA fragments were amplified by PCR using primers pV-M-F and m3-F1-R (Table A1), and plasmid pcVd380-380, pcVm1d380-380, pcVm2d380-380 or pcVm12d380-380 as templates. A 1024-bp DNA fragment was amplified by PCR using primers m3-F2-F and pV-XhoI-R (Table A1), and plasmid pcV.d3 as a template. In the third PCR, each fragment from the first PCR and the fragment from the second PCR were annealed and used to amplify four 2043-bp DNA fragments by overlapping PCR using primers pV-M-F and pV-XhoI-R. Each 1144-bp *Eco*RI-*Xho*I DNA fragments of these four 2043-bp fragments were ligated to *Eco*RI-*Xho*I digested plasmid pcDNA3 to create plasmid pcV.m3d3, pcV.m13d3, pcV.m23d3 and pcV.m123d3, respectively.

j) Plasmid pcV.d4.A 1157-bp DNA fragment was amplified by PCR using primers pV-EcoRI-F and d(380-423)XhoI-R (Table A1), and pcV as a template. A 1143-bp *Eco*RI-*Xho*I fragment of the 1157-bp fragment was isolated and ligated to *Eco*RI-*Xho*I digested pcDNA3 to construct pcV.d4.

k) Plasmid pcV.d5 and pcV.d6. Two 995-bp and 842-bp DNA fragments were amplified by PCR using primers pV-EcoRI-F and d(323-423)XhoI-R (Table A1), and pcV and pcV.d1d2 as template, respectively. 883-bp and 828-bp *Eco*RI-*Xho*I DNA fragments of the 995-bp and 842-bp DNA fragment, respectively, were ligated to the *Eco*RI-*Xho*I digested pcDNA3 to create pcV.d5 and pcV.d6, respectively.

l) Plasmid pcV.d21-50/380-423, pcV.d18 and pcV.d12. A 445-bp *Age*I-*Xho*I fragment of pcV.d4 was isolated and ligated to *Age*I-*Xho*I digested pcV.d1, pcV.d2 and pcV.d1d2 to create *pcV.d21-50/380-423,* pcVd18 and pcV.d12, respectively.

m) Plasmid pcVd10. A 905-bp DNA fragment was amplified by PCR using primers d(1-100)EcoRI-F and pV-XhoI-R (Table A1), and pcV.d18as template. A 891-bp *Eco*RI-*Xho*I band of the 905-bp fragment was ligated to *Eco*RI-*Xho*I digested pcDNA3-HA to construct pcV.d10.

n) Plasmid pcV.d9. A 1130-bp DNA fragment was amplified by PCR using primers pV-M-F and d(101-210)-F1-R (Table A1), and plasmid pcV.d21-50/380-423 as a template. A 648-bp DNA fragment was amplified by PCR using primers d(101-210)-F2-F and d(380-423)XhoI-R (Table A1), and plasmid pcVd21-50/380-423 as template. In the third PCR, two PCR fragments were annealed and used to amplify a 1733-bp DNA fragment without by overlapping PCR using primers pV-M-F and d(380-423)XhoI-R. An 834-bp *Eco*RI-*Xho*I DNA fragment of the 1733-bp fragment was ligated to *Eco*RI-*Xho*I digested plasmid pcDNA3 to create plasmid pcV.d11.

o) Plasmid pcV.d7 and pcV.d8. Two 590-bp and 500-bp fragments were amplified by PCR using primers pV-EcoRI-F and d(190-423)XhoI-R (Table A1), and pcV and pcV.d1, respectively, as templates. 576-bp and 486-bp *Eco*RI-*Xho*I bands of the 590-bp and 500-bp fragments, respectively, were ligated to *Eco*RI-*Xho*I digested pcDNA3 to construct pcV.d7 and pcV.d8, respectively.

p) Plasmid pcV.d15 and pcV.d11. Two 1156-bp and 1066-bp DNA fragments were purified by PCR using primers M-F and d(81-120)-F1-R (Table A1), and pcV and pcV.d1, repectively, as templates. An 853-bp DNA fragment was amplified by PCR using primers d(81-120)-F2-F and pV-d(380-423)XhoI-R (Table A1), and plasmid pcV.d18 as a template. In the third PCR, each fragment from the first PCR and the fragment from the second PCR were annealed and used to amplify 1967-bp and 1877-bp fragments by overlapping PCR using primers pV-M-F and pV-d(380-423)XhoI-R. 1099-bp and 1009-bp *Bam*HI-*Xho*I DNA fragments of the 1967-bp and 1877-bp fragments, respectively, were ligated to *Bam*HI-*Xho*I digested plasmid pcDNA3 to create plasmid pcV.d15and pcVd11, respectively.

q) Plasmid pcV.d13 and pcV.d14.A 641-bp *Sna*BI DNA fragment from plasmid pcV.d11 was isolated and ligated to *Sna*BI digested plasmid pcV.d4 and pcV.d2 to create pcV.d13 and pcVd14, respectively.

r) Plasmid pcV.d19.A 582-bp *Age*I-*Xho*I DNA fragment from plasmid pcV.d15 was isolated and ligated to *Age*I-*Xho*I digested pcV to create pcV.d15.

s) Plasmid pcV.d17.A 731-bp *Sna*BI fragment of plasmid pcV.d15 was isolated and ligated to *Sna*BI digested pcV.d4 to construct pcV.d17.

t) Plasmid pcV.d16.A 1895-bp DNA fragment was amplified by PCR using primers M-F and d(390-423)XhoI-R, and plasmid pcV.d19 as a template. A 1027-bp *Bam*HI-*Xho*I band of the 1895-bp fragment was ligated to *Bam*HI-*Xho*I digested pcDNA3 to create pcV.d16.

u) Plasmid pNoLS1.EY. A 112-bp DNA fragment was purified by PCR using primers NoLS1-EY-F and NoLS1-EY-R (Table A1), and pcV as a template. A 98-bp *Bam*HI-*Sal*I band of the 112-bp fragment was ligated to *Bam*HI-*Sal*I digested pEYFP-N1 to construct pNoLS1.EY.

v) Plasmid pNoLS2.EY. A 1237-bp DNA fragment was amplified by PCR using primers NoLS2-EY-F and NoLS2-EY-R (Table A1), and plasmid pEYFP-N1 as a template. A 772-bp *Bam*HI-*Sal*I band of the 1237-bp fragment was ligated to *Bam*HI-*Sal*I digested pEYFP-N1 to create pNoLS2.EY.

w) Plasmid pNoLS1-GFPβGal. A 116-bp DNA fragment was amplified by PCR using primers GB-NoLS1-BamHI-F and GB-NoLS1-NheI-R (Table A1), and plasmid pcV as a template. A 102-bp *Bam*HI-*Nhe*I band of the 116-bp fragment was isolated and ligated to *Bam*HI-*Nhe*I digested pCMVGbeta to create pNoLS1-GFPβGal.

References:

Depping, R., Steinhoff, A., Schindler, S.G., Friedrich, B., Fagerlund, R., Metzen, E., Hartmann, E., Kohler, M., 2008. Nuclear translocation of hypoxia-inducible factors (HIFs): involvement of the classical importin alpha/beta pathway. Biochimica et biophysica acta 1783, 394-404.

Du, E., Tikoo, S.K., 2010. Efficient replication and generation of recombinant bovine adenovirus-3 in nonbovine cotton rat lung cells expressing I-SceI endonuclease. The journal of gene medicine 12, 840-847.

Lai, M.C., Lin, R.I., Tarn, W.Y., 2001. Transportin-SR2 mediates nuclear import of phosphorylated SR proteins. Proceedings of the National Academy of Sciences of the United States of America 98, 10154-10159.

Wu, Q., Chen Y, Kulshrestha V, Tikoo, SK. 2004.Characterization and nuclear localization of fiber protein encoded by thew late region L7 of bovine adenovirus-3. Arch. Virol. 149:1783-1789 *99*, 9-15.

Table A1. List of primers

Name	Sequence
pV-EcoRI-F	5'- GGAGCCGAATTCATGGCCTCCTCTCGGTTGATTAAAGAAG
M-F	5- TCTGCTCTGA TGCCGCATAGTTAAGCC
d(21-50)-F1-R	5- CGCTTTCTAGAGCCGCGGTAAATCTCAGGCGCCACGA TGTC
d(21-50)-F2-F	5-TCGTGGCGCCTGAGATTTACCGCGGCTCTAGAAAGCGGG CCTTG
pV-XhoI-R	5-AATACTCGAGAGCGCTTAACGGCGGAGCCGGGTTAC
d(190-210)-F1-R	5'-GGTCATCCTCATTCTCGGCCATGGAAAGAATAACTTTCTGCTCGG
d(190-210)-F2-F	5'-CCGAGCAGAAAGTTATTCTTTCCATGGCCGAGAATGAGGATGACCG
d(380-389) F1-R	5'-CAGCGCTGAGGCGGGGAGTCGCGACTGCAGGCAGGCGCACAC
d(380-389) F2-F	5'-GTGTGCGCCTGCCTGCAGTCGCGACTCCCCGCCTCAGCGCTG
d(380-389)-F2-R	5'- GTGGCGAGAAAGGAAGGGAAGAAAG
m1-F1-R	5'CGCGTTCTCGCCTGGGTGCAGCTCCTGCGTAGATCTCAGGCGCCACG ATGTC
m1-F2-F	5'TGCGCCGGGTTTTGCGTCCCAGATCTATGGCCGAGAATGAGGATGAC C
m2-F1-R	5'CATATGGTGCTGCTGCTGCTGCTGCGGGCCGTTTGCGCTTGTAAATCT C
m2-F2-F	5'AAGCGCAAACGGCCCGCAGCAGCAGCAGCAGCACCATATGCTGTGA AG CAGGAG
M12-F1-R	5-CTGCAGCAGCTGCTGCGGGTGCAGCTCCTGCGTAAATCTCAGGCGC CACGATG
M12-F2-F	5-CGCAGGAGCTGCACCCGCAGCAGCTGCTGCAGCACCGTATGCTGTG AAG
M3-F1-R	5-TTTCTAGAGCCGCGAGCAGCTGCTGCCTCCGCCTTTACTAAAGGCTT CTC
M3-F2-F	5-TTAGTAAAGGCGGAGGCAGCAGCTGCTCGCGGCTCTAGAAAGCGG GCCTTG
d(380-423)XhoI-R	5'-TAATCTCGAGTTACCTGCGGGTACGGCGGCGACGG
pV-d(323-423)XhoI-R	5'- GATCCTCGAGTCAGTCAGTTTGGGTGCTGAGTG
d(1-100)EcoRI-F	5'-CGATGAATTCATGGGCGTGGCCGTTAGTTTCAC
d(101-210)-F1-R	5'- CATCCTCATTCTCGGCCATAGATCTGGGACGCAAAACCCGGCGCAC C
d(101-210)-F2-F	5'- TGCGCCGGGTTTTGCGTCCCAGATCTATGGCCGAGAATGAGGATGA CC
d(190-423)XhoI-R	5'- GATACTCGAGTTAGCGCCGCTTCTTGCTAGCTAAAACC
dV-F2-R	5- GTCC-ATGGCGTGTTAACAAGCTGTG
d(81-120)-F1-R	5'- GTCTGCGTACACCTCGTCATACACGAATTCTATGTGGGGCTCGTCG
d(81-120)-F2-F	5'- GAGCCCCACATAGAATTCGTGTATGACGAGGTGTACGCAGAC
d(390-423)XhoI-R	5'- TAATCTCGAGATGGCGACTCCCCGCCTCAGCGCTGC
NoLS1-EY-F	5'-TTAGGTCGACATGAAGCGCAAACGGCCCAGGCGAGAAC
NoLS1-EY-R	5'-GCACGGATCCTTAATTTTGCGCTCCGCCTTTAC
NoLS2-EY-F	5'-TTAAGGATCCTATGCGGCGCCGCCGTCGCCGCCGTACCCGCAGGAT GGTGAGCAAGGGCGAGGAGCTGTTC
NoLS2-EY-R	5'-AGTGGGCCATCGCCCTGATAGAC
GB-NoLS1-BamHI-F	5'-TAATGGATCCATGAAGCGCAAACGGCCCAGGCGAGAAC
GB-NoLS1-NheI-R	5'-CTTTGCTAGCCATCTTAATTTTGCGCTCCGCCTTTAC
